# Obtaining EQ-5D-5L utilities from the disease specific quality of life Alzheimer’s disease scale: development and results from a mapping study

**DOI:** 10.1007/s11136-020-02670-8

**Published:** 2020-10-17

**Authors:** Ines Rombach, Marvi Iftikhar, Gurleen S. Jhuti, Anders Gustavsson, Pascal Lecomte, Mark Belger, Ron Handels, Amparo Y. Castro Sanchez, Jan Kors, Louise Hopper, Marcel Olde Rikkert, Geir Selbæk, Astrid Stephan, Sietske A. M. Sikkes, Bob Woods, Manuel Gonçalves-Pereira, Orazio Zanetti, Inez H. G. B. Ramakers, Frans R. J. Verhey, John Gallacher, Actifcare Consortium, LeARN Consortium, Filipa Landeiro, Alastair M. Gray, ROADMAP Consortium

**Affiliations:** 1 Nuffield Department of Population Health, Health Economics Research Centre, University of Oxford, Old Road Campus, Oxford, OX3 7LF United Kingdom; 2 Global Access, Centre of Excellence F.Hoffmann-La Roche Ltd, CH-4070 Basel, Switzerland; 3 Quantify Research, Stockholm, 112 21 Sweden; 4 Division of Neurogeriatrics, Department for Neurobiology, Care Sciences and Society, Karolinska Institutet, Solna, 171 64 Sweden; 5 Global Head Health Economic Modelling and Methodology, Novartis Pharma AG, 4002 Basel, Switzerland; 6 Global Statistical Sciences, Eli Lilly and company, Erl Wood Manor, Windlesham, GU20 6PH United Kingdom; 7 Faculty of Health, Medicine and Life Sciences, School for Mental Health and Neuroscience, Department of Psychiatry and Neuropsychology, Alzheimer Center Limburg, Maastricht University Medical Center, Maastricht, 6200 MD The Netherlands; 8 Department of Medical Informatics, Erasmus MC-University Medical Center Rotterdam, Rotterdam, 3015 GD The Netherlands; 9 School of Psychology, Dublin City University, Dublin 9, Ireland; 10 Department of Geriatrics, Radboudumc Alzheimer Center, Donders Center for Medical Neuroscience, Radboud University Medical Center, Nijmegen, 6525 GA The Netherlands; 11 National Advisory Unit of Ageing and Health, Vestfold Hospital Trust, 3103 Tønsberg, Norway; 12 Department of Geriatric Medicine, Oslo University Hospital, Oslo, 0372 Norway; 13 Faculty of Medicine, University of Oslo, Oslo, 0372 Norway; 14 Institute for Health and Nursing Science, Martin Luther University Halle-Wittenberg, Halle (Saale), 06112 Germany; 15 Alzheimer Center Amsterdam, Amsterdam University Medical Centers/Amsterdam Neuroscience, Amsterdam, 1007 MB The Netherlands; 16 Dementia Services Development Centre Wales (DSDC), Bangor University, Bangor, LL57 2PZ United Kingdom; 17 Nova Medical School/Faculdade de Ciências Médicas, Universidade Nova de Lisboa, Lisbon, 1169-056 Portugal; 18 CHRC (Comprehensive Health Research Centre), Lisbon, Portugal; 19 IRCCS Istituto Centro San Giovanni di Dio Fatebenefratelli, Brescia, 25125 Italy; 20 Dementias Platform UK, Department of Psychiatry, University of Oxford, Warneford Hospital, Oxford, OX3 7JX United Kingdom

**Keywords:** Mapping algorithm, Cross-walking, Health related quality of life, Preference based measures, Dementia

## Abstract

**Purpose:**

The Quality of Life Alzheimer’s Disease Scale (QoL-AD) is commonly used to assess disease specific health-related quality of life (HRQoL) as rated by patients and their carers. For cost-effectiveness analyses, utilities based on the EQ-5D are often required. We report a new mapping algorithm to obtain EQ-5D indices when only QoL-AD data are available.

**Methods:**

Different statistical models to estimate utility directly, or responses to individual EQ-5D questions (response mapping) from QoL-AD, were trialled for patient-rated and proxy-rated questionnaires. Model performance was assessed by root mean square error and mean absolute error.

**Results:**

The response model using multinomial regression including age and sex, performed best in both the estimation dataset and an independent dataset.

**Conclusions:**

The recommended mapping algorithm allows researchers for the first time to estimate EQ-5D values from QoL-AD data, enabling cost-utility analyses using datasets where the QoL-AD but no utility measures were collected.

**Electronic supplementary material:**

The online version of this article (10.1007/s11136-020-02670-8) contains supplementary material, which is available to authorized users.

## Background

Dementia, a progressive neurodegenerative syndrome, is increasing in prevalence due to more people living into the age-groups most at risk and predicted to affect around 131 million people globally by 2050 [1]. It is characterised by cognitive and functional decline, including memory loss, communication problems, behavioural changes, and deterioration in the ability to carry out activities of daily living [2] that can impact on the quality of life (QoL) of people living with dementia and their carers [3].

QoL is defined by the WHO as ‘an individual’s perception of their position in life in the context of the culture and value systems in which they live and in relation to their goals, expectations, standards and concerns’ [4]. Health-related quality of life (HRQoL), reflects ‘the individual’s perception of the impact of a health status, on the ability to perform usual tasks and effects on everyday life, and on physical, social and emotional well-being’ [5].

Disease specific HRQoL instruments, such as the Quality of Life Alzheimer’s Disease scale (QoL-AD) [6] and the DEMQOL [7], are commonly used in research and may be more sensitive in detecting effects of interventions [8, 9]. However, to assess cost-effectiveness across conditions, a generic utility-based instrument such as the EQ-5D is often required [10]. Where research using only disease-specific instruments has been performed but cost-effectiveness analysis is desired, mapping algorithms can be used to estimate EQ-5D outcomes.

Although preference based indices for use in economic evaluation exist for the DEMQOL [7, 11, 12], these are not available for the QoL-AD, the most commonly used and foremost recommended disease-specific questionnaire in studies of people with dementia [13-17]. Here we report the development and validation of a mapping algorithm from the QoL-AD to the EQ-5D, which has not previously been undertaken, according to a review of published mapping studies [18].

This study forms part of the Real world Outcomes across the AD spectrum for better care: Multi-modal data Access Platform (ROADMAP) project.

## Methods

This mapping study was performed in line with recommendations from the MAPS statement [19], which consolidates best practice for mapping studies, including dataset selection, modelling and analysis, and reporting [20].

### Data sources

We used data from the Actifcare study [21], a longitudinal cohort study aiming to develop best practice for access to formal care for persons with dementia in the community. Participants (n = 451) were recruited between 2014 and 2016 through memory clinics, general practices, case managers and community mental health teams in eight European countries (Germany, Netherlands, Sweden, Norway, Ireland, United Kingdom, Portugal and Italy). Eligible participants had to have a diagnosis of dementia meeting DSM IV TR criteria following an assessment by a clinical professional and have a Clinical Dementia Rating (CDR) of 1 or 2, or a Mini-Mental State Examination (MMSE) score of 24 or below. Eligible participants had an informal carer, but no regular assistance from a paid worker for their personal care due to their dementia, and were likely to require formal assistance over the next year [21].

Data on the EQ-5D-5L and the QoL-AD for the person with dementia were collected at baseline, 6 and 12 months, rated independently by both people with dementia (self-rated) and their informal carers on behalf of people with dementia (proxy-rated). Data collection was interview based and performed by trained researchers.

The clinical cohort study of the LeARN project [22] was used as an external validation dataset. The study, conducted in the Netherlands, recruited 241 patients who visited a memory clinic for the evaluation of cognitive complaints. They had to meet the inclusion criteria of being suspected of having a primary neurodegenerative disease (without a formal diagnosis), MMSE of at least 20, and a CDR of 0-1 to reflect current clinical practice and enable generalisability. HRQoL outcomes, including the QoL-AD (proxy-rated only) and EQ-5D-3L (self-scored and proxy-rated), were collected. Data for this validation study were collected at baseline, 12 and 24 months. Patient self-scored EQ-5D data were collected through interviews with research nurses in three memory clinics, proxy-rated data were self-administered.

### HRQoL instruments

The QoL-AD questionnaire consists of 13 items covering overall HRQoL, relationships with family and friends, physical health, memory, and ability to perform household chores and activities. Each item has four possible response levels (poor, fair, good and excellent), scored from 1 to 4, respectively. A composite QoL-AD score between 13 and 52 is calculated by adding up the items, with higher scores representing higher HRQoL. Item 7 is related to marriage, which may not apply to those who identify themselves as widowed, single, separated or divorced. For this reason, unless stated otherwise, in this work item 7 is not included in the total score, which is standardised to maintain a range from 13 to 52. Originally developed in the English language (US), most of the available translations of the QoL-AD were performed by the linguistic validation company Mapi/ICON Language Services, withfull details available from the developers [23]. Cross-cultural validations have been performed for some countries, including Portugal, Germany, France, the Netherlands, the UK and Sweden [24-28]. For the Actifcare study, a translation protocol was used for questionnaires not available in all languages [21].

The EQ-5D-5L has five items (domains) covering mobility, self-care, usual activities, pain/discomfort, and anxiety/depression [29, 30]. Each item has five response levels: no problems, slight problems, moderate problems, severe problems, unable to perform activity or extreme problems. A validated UK value set is still being developed for the EQ-5D-5L, and so, in line with current NICE recommendations, we use a “cross-walk” developed by van Hout et al to the existing EQ-5D-3L utilities [31]. The EQ-5D generates generic, preference based utilities which reflect the strength of preference of the general population for different health states. Utilities have a maximum value of one, indicating perfect health. Zero indicates a health state equal to death and negative values represent states considered worse than death.

For the purposes of this mapping study, the UK value set was applied to all EQ-5D data.

### Statistical methods

Observations with non-missing data for all relevant QoL-AD and EQ-5D items were included in the mapping exercise.

All data exploration and statistical models were applied separately to the different mapping scenarios. We first explored the relationship between the two measures visually using scatter plots and cross-tabulations, as well as Spearman’s correlation coefficients, then trialled a range of statistical models.

Direct mapping describes models where the explanatory variables (here: the QoL-AD items or scores) are directly mapped onto the EQ-5D utility score. We used ordinary least squares (OLS) regression, Tobit, centred least absolute deviation (CLAD) and two-part models, in which logistic regression is used to predict whether participants were in perfect health, and an OLS model to predict utilities for participants not in perfect health. Tobit, CLAD and two-part models are able to account for the ceiling effect in the EQ-5D utilities, i.e. clustering of responses at the maximum score of 1, which represents perfect health. Predicted values greater than 1 were set to 1 for the other scenarios.

Response models used the explanatory variables to predict responses to each individual EQ-5D question, then combined the five question responses to obtain utilities. As each question was modelled separately, each response mapping algorithm consisted of five separate models. Responses to the items were predicted using OLS, multinomial logistic regression (mlogit) and ordinal logistic regression (ologit).

QoL-AD items were used as categorical variables in all models except the ‘continuous OLS’ model which mapped QoL-AD composite scores directly to the EQ-5D utilities, and the ‘Response OLS Continuous’ model, which used each QoL-AD item as a continuous variable. To account for clustering of observations within participants, the ‘cluster’ option in Stata was used in all models except the CLAD. The models presented in the manuscript did not include QoL-AD item 7; results including QoL-AD item 7 can be found in the supplemental material.

All models were run as described above, and repeated including age of the person with dementia at time of assessment (as a continuous variable) and sex (as a categorical variable). Including age as a non-linear explanatory variable was also explored.

Utilities for the two-part model were calculated as follows:

$${\text{Utility}} = {\Pr}\left( {{\text{PerfectHealth}}} \right) + \left( {{1} - {\Pr}\left( {{\text{PerfectHealth}}} \right)} \right) \, \times {\text{ Y}}$$

where Pr(PerfectHealth) is the predicted probability that utility = 1 and Y = predicted utility conditional on imperfect health [32].

Predicted responses to the EQ-5D-5L were cross-walked to the EQ-5D-3L, and utilities were then derived from those.

The prediction accuracy of the models was explored by comparing root mean square errors (RMSE) and mean absolute errors (MAE) across different centiles of the population. Visual comparisons in the form of scatter plots showing observed versus predicted EQ-5D utilities, and comparisons of predicted responses to each question against the values observed, were performed to gauge how prediction accuracy varied across patient characteristics.

### External validation of the mapping algorithm

The different mapping algorithms were also trialled in the external validation dataset, to establish which models performed best in an independent dataset. The recommended mapping algorithm was applied to the validation dataset to assess how well these mapping algorithms were able to predict observed EQ-5D utilities in this dataset independent of the algorithm development process.

We aimed to estimate EQ-5D utilities from observed QoL-AD data, and the main text focuses on:

- Mapping self-rated QoL-AD to self-rated EQ-5D-5L.
- Mapping proxy-rated QoL-AD to proxy-rated EQ-5D-5L.

For completeness, additional scenarios are reported in the supplementary material:

- Mapping self-rated QoL-AD to proxy-rated EQ-5D-5L.
- Mapping proxy-rated QoL-AD to self-rated EQ-5D-5L.

We put less emphasis on these last two scenarios due to concerns in the literature about discrepancies between self-rated and proxy-rated health states in this patient population [25, 33].

All statistical programming was performed in Stata/SE (StataCorp. LP, College Station, Texas).

## Results

### Description of the population

Table 1 and Supplemental Table 1 show the characteristics of the study populations and correlations between the QoL-AD and EQ-5D. Supplemental Table 1 shows responses to each EQ-5D-5L and QoL-AD question, while Supplemental Table 3 contrasts the characteristics of participants whose data were included in and excluded from the mapping study.

**Table 1:** Overview of the data used

Demographic variable	Estimation dataset		Validation dataset
	Self-rated QoL-AD → Self-rated EQ-5D	Proxy-rated QoL-AD → Proxy- rated EQ-5D	Proxy-rated QoL-AD → Proxy- rated EQ-5D
Total number of observations in datasets	1353	1353	753
Total number of observations excluded from analysis*	333 (25%)	254 (19%)	122 (16%)
Total number of observations included in analysis	1020 (75%)	1099 (81%)	631 (84%)
Total number of additional observations excluded if QoL-AD item 7 was included in analysis	137/1020 (13%)	156/1099 (14%)	72/631 (11%)
Number of participants included in analysis	427	437	235
PwD age (SD)	78 (8)	78 (8)	67 (9)
Proxy age (SD)	66 (13)	67 (13)	62 (11)
PwD sex (female)	55%	54%	34%
Proxy sex (female)	67%	67%	73%
MMSE**	19 (5)	19 (5)	25 (4)
CDR 0***	0%	0%	11%
CDR 0.5***	3%	3%	52%
CDR 1***	70%	67%	32%
CDR 2***	26%	28%	5%
CDR 3***	1%	3%	< 1%
Self-rated QoL-AD mean (SD)	35 (6)	n/a	n/a
Self-rated QoL-AD median (range)	36 (16, 52)	n/a	n/a
Proxy-rated QoL-AD mean (SD)	n/a	30 (6)	32 (5)
Proxy-rated QoL-AD median (range)	n/a	30 (15, 50)	31 (15, 52)
Self-rated EQ-5D Utility mean (SD)	0.77 (0.21)	n/a	n/a
Self-rated EQ-5D Utility median (range)	0.81 (− 0.26, 1)	n/a	n/a
Proxy-rated EQ-5D Utility mean (SD)	n/a	0.60 (0.24)	0.77 (0.22)
Proxy-rated EQ-5D Utility median (range)	n/a	0.64(-0.31, 1)	0.81(− 0.10, 1)
Spearman’s Correlation (95% CI)	0.49 (0.45, 0.54)	0.48 (0.43, 0.52)	0.56(0.50, 0.61)

*CI* confidence interval, *PwD* person with dementia, *SD* standard deviation

*Insufficient EQ-5D-5L or QOL-AD data were available for inclusion in the mapping study, either through unavailability of the complete questionnaire, or individual items

**MMSE (Mini-mental state examination) data were unavailable for a proportion of people with dementia. the following percentage of the total number of observations are excluded from the MMSE summaries: Estimation dataset:8% in the ‘Self-rated QoL-AD Self-rated EQ-5D’ scenario; 13% in the ‘Proxy-rated QoL-AD Proxy- rated EQ-5D’ scenario ; Validation dataset: 32%. The score ranges from 0 to 30, with higher scores indicating less cognitive impairment

***CDR (clinical dementia rating) data were unavailable for a proportion of observations; the following percentage of the total number of observations are excluded from the CRD summaries: estimation dataset: 1% in the ‘Self-rated QoL-AD Self-rated EQ-5D’ scenario; 2% in the ‘Proxy-rated QoL-AD Proxy- rated EQ-5D’ scenario ; validation dataset: 38%. Missing data occurred predominantly due to CDR assessments not being performed, rather than individual domains of cognitive and functional performance being missing. The percentages presented are based on the population with available CDR data only

Four-hundred-and-fifty-one persons with dementia (PwD) were included in the estimation dataset, with 1353 intended observations (up to three time points for each participant). Sufficient data for 427 to 437 participants (1017 to 1099 observations) were available for inclusion in the different mapping scenarios. 631 observations from 235 participants were used from the main validation dataset.

The summaries presented in Table 1 (and Supplemental Table 1) contain all observations included in the relevant analyses to provide an overview of the data used in the mapping study; participants may be included multiple times to reflect data collected at different follow-up time points.

PwD in the estimation dataset were on average 11 years older than those in the validation dataset. There were similar amounts of male and female PwD in the estimation dataset, while PwD in the validation dataset were predominantly male. Carers in both datasets were predominantly female. PwD showed higher levels of cognitive decline using the MMSE and CDR in the estimation dataset, compared to the validation dataset, in line with the inclusion criteria of the two studies. Similar trends were observed in the QoL-AD and EQ-5D scores, ith PwD providing higher scores than the proxy-ratings by their carers.

Spearman’s correlation values close to 0.5 were observed between QoL-AD scores and EQ-5D utilities were observed in the validation dataset; corresponding correlations were higher in the validation dataset.

Scatter plots (Supplemental Fig. 1) show the variation in observed EQ-5D utilities for observed QoL-AD scores. Correlations between the individual QoL-AD and EQ-5D items ranged from close to 0 to -0.53 in the validation dataset; correlations were higher where both questionnaires were either self-rated or proxy-rated, and in the validation dataset (Supplemental Table 4).

Table 1 and Supplemental Table 1 demonstrate that between 11% and 15% of participants have additional missing data for QoL-AD item 7.

### Comparison of the mapping algorithms

Nine different mapping algorithms were evaluated using the Actifcare dataset for each of the different scenarios of self-rated and proxy-rated questionnaires.

Table 2 shows the performance parameters (RMSE and MAE) for each algorithm, with age and sex included.

**Table 2:** Comparison of mapping algorithms (including age and sex)

Model	Self-rated QoL-AD → Self-rated EQ-5D					Proxy- rated QoL-AD → Proxy- rated EQ-5D				
	RMSE	MAE	Minimum predicted score*	Maximum predicted score*	Accuracy within 0.1 points (%)	RMSE	MAE	Minimum predicted score^+^	Maximum predicted score+	Accuracy within 0.1 points (%)
Direct OLS Continuous	0.1797	0.1302	0.460	1.000	49	0.2109	0.1614	0.336	1.000	42
Direct OLS Categorical	0.1614	0.1196	0.249	1.000	51	0.1916	0.1473	0.233	1.000	44
Direct Tobit	0.1612	0.1191	0.233	0.974	52	0.1915	0.1471	0.229	0.958	45
Direct Clad	0.1677	0.1195	0.293	1.000	54	0.2012	0.1497	0.200	1.000	45
Direct 2-part	0.1610	0.1192	0.259	0.982	52	0.1913	0.1465	0.241	0.986	45
Response OLS Categorical	0.1765	0.1267	0.378	1.000	49	0.2062	0.1555	0.378	1.000	42
Response OLS Continuous	0.1913	0.1386	0.533	1.000	45	0.2140	0.1633	0.393	1.000	40
Response ologit	0.1624	0.1196	0.210	0.964	51	0.1928	0.1491	0.172	0.921	43
Response mlogit	**0.1348**	**0.1063**	-0.253	0.972	53	**0.1819**	**0.1401**	-0.051	0.930	47

The lowest root mean square error (RMSE) and mean absolute error (MAE) are highlighted in bold

*The observed minimum and maximum self-rated EQ-5D scores were − 0.261 and 1, respectively

^**+**^The observed minimum and maximum proxy-rated EQ-5D scores were − 0.307 and 1, respectively

The response mapping using mlogit models was found to produce the lowest RMSEs and MAEs for both scenarios.

Some convergence issues were observed when estimating the CLAD, ologit and mlogit models. The maximum iterations run were set to 200 (400 for CLAD), and coefficient estimates obtained at this point were used in the mapping algorithm.

Performance parameters for the corresponding models excluding age and sex, and when including QoL-AD item 7, age and sex are shown in Supplemental Tables 5. RMSEs and MAEs were approximately 5% larger when age and sex were excluded as explanatory variables.

The mlogit model including QoL-AD item 7 produced marginally smaller standard errors compared to the model excluding QoL-AD item 7 (differences of up to 2% for RMSE and MAE), but could only be run on a subset of the data available for the other models (Table 1).

In some instances, the number of respondents reporting particular question responses was small, and perfect prediction of actual EQ-5D responses resulted in questionable standard errors and coefficients for some of the mlogit and ologit models. To address this issue, results for the direct Tobit model are also presented in the supplemental material.

### Predictive accuracy of the mlogit model

**Fig. 1:**
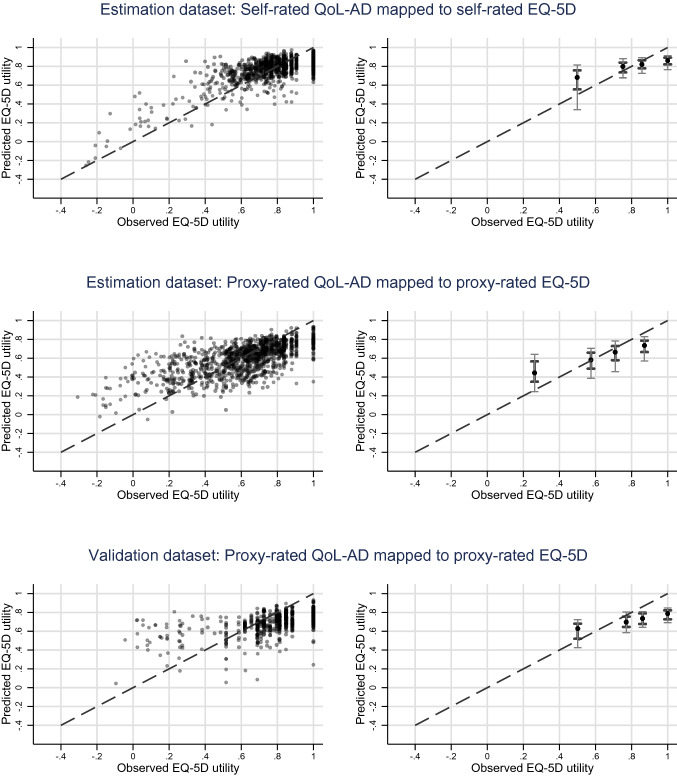
Prediction accuracy of the preferred (mlogit) mapping model. Note: Scatter plots of predicted versus observed utilities are presented in the left-hand column. Darker markers on the graphs indicate overlapping data points. Observed utilities have been classed into quartiles in the right-hand column, and the means of these quartiles are shown on the* x*-axis. On the* y*-axis, the median, interquartile range (thicker, darker vertical lines) and 10th to 90th centiles (thinner, lighter vertical lines) of the predicted utilities are shown on the* y*-axis to represent the data distribution

Figure 1 illustrates the prediction accuracy of the preferred response mapping model (using mlogit, including age and sex but excluding QoL-AD question 7), showing observed EQ-5D utility against predicted EQ-5D utility for the self-reported to self-reported and proxy-reported to proxy-reported scenarios. Corresponding plots for other scenarios and the Tobit model are shown in Supplemental Fig. 3. Perfect agreement between observed and predicted values is indicated by the dashed line. The plots demonstrate variation in the predicted values for a given observed EQ-5D utility score, and the mapping algorithm does not predict utilities of 1 in any of the scenarios. Generally, the model has a tendency to over-predict for low observed EQ-5D utilities, and to under-predict for higher levels of observed EQ-5D utilities.

Similar patterns were observed for all mapping scenarios examined, with higher prediction accuracy when QoL-AD scores were above the median (Supplemental Table 6).

The mlogit model generates probabilities of an individual falling into each of the five levels for each question of the EQ-5D-5L. Supplemental Figure 3 shows the mean probabilities of falling into each of the five domains, given their observed response to each item. The model generally predicts well for those indicating no or extreme problems. For those with slight, moderate and severe problems, the model tends to predict lower levels of problems than observed. Predictions were more accurate when mapping either between self-rated or between proxy-rated data.

### Validation

Trialling the different mapping approaches in the external validation study confirmed that the mlogit response mapping model resulted in the smallest RMSEs and MAEs (Supplemental Table 3d); RMSEs and MAEs of 0.2000 and 0.1591 respectively were produced when the preferred mapping model derived from the estimation dataset was applied to the validation dataset for the scenario mapping proxy-reported QoL-AD to proxy-reported EQ-5D. As in the estimation dataset, predictions in the validation exercise tended to over-predict for utilities below 0.7 and under-predict for utilities above 0.7 (Fig. 1, Supplemental Table 6).

### Predicted utilities in relation to observed QoL-AD scores

Table 3 shows actual QoL-AD responses for some individual participants in the estimation dataset. It demonstrates how different combinations of responses to the QoL-AD items can result in identical QoL-AD composite scores. As the mapping algorithm is based on responses to individual QoL-AD items, different utilities were generated for participants with the same total QoL-AD score. The relationship between QoL-AD items and EQ-5D items also differs between the different mapping scenarios, resulting in different utilities being allocated to the same combination of QoL-AD items depending on whether proxy-rated or self-rated data are used. Ranges of predicted utilities for selected observed composite QoL-AD scores are shown in Supplemental Table 7, and the range of predicted and observed EQ-5D utilities for every observed QoL-AD score are shown in Supplemental Figure 4. Despite the issues illustrated in Table 3, there is a clear trend whereby higher observed QoL-AD scores result in higher predicted utilities.

**Table 3:** Mapped EQ-5D utilities for observed QoL-AD scores

	Responses	QoL-AD score of ~25			QoL-AD score of ~35			QoL-AD score of 45		
		Participant #1	Participant #2	Participant #3	Participant #4	Participant #5	Participant #6	Participant #7	Participant #8	Participant #9
Observed data	QoL-AD Item 1	Fair	Fair	Fair	Fair	Good	Excellent	Good	Excellent	Good
	QoL-AD Item 2	Fair	Poor	Fair	Poor	Fair	Good	Good	Excellent	Excellent
	QoL-AD Item 3	Good	Poor	Fair	Good	Good	Fair	Excellent	Excellent	Good
	QoL-AD Item 4	Poor	Fair	Good	Excellent	Good	Good	Excellent	Good	Excellent
	QoL-AD Item 5	Poor	Poor	Fair	Fair	Fair	Poor	Good	Poor	Excellent
	QoL-AD Item 6	Poor	Fair	Good	Excellent	Good	Excellent	Excellent	Excellent	Excellent
	QoL-AD Item 8	Good	Fair	Poor	Good	Fair	Good	Excellent	Good	Excellent
	QoL-AD Item 9	Fair	Fair	Fair	Good	Good	Excellent	Good	Excellent	Good
	QoL-AD Item 10	Poor	Fair	Fair	Poor	Fair	Fair	Good	Good	Good
	QoL-AD Item 11	Fair	Good	Fair	Good	Good	Good	Good	Excellent	Excellent
	QoL-AD Item 12	Good	Fair	Poor	Good	Good	Poor	Excellent	Excellent	Good
	QoL-AD Item 13	Fair	Good	Poor	Good	Good	Fair	Excellent	Excellent	Good
	Age	92	84	79	85	81	57	85	86	69
	Sex	Male	Male	Female	Female	Male	Male	Female	Female	Male
	Sum of QoL-AD items	23	23	23	32	32	32	42	42	42
	Standardised QoL-AD*	24.9	24.9	24.9	34.7	34.7	34.7	45.5	45.5	45.5
Mapped EQ-5D utility (UK value set)	Self-rated QoL-AD → Self-rated EQ-5D	0.430	0.665	0.780	0.551	0.810	0.898	0.820	0.902	0.919
	Proxy-rated QoL-AD → Proxy-rated EQ-5D	0.414	0.570	0.591	0.381	0.688	0.838	0.783	0.874	0.769

*QoL-AD score is standardised to range from 13 to 52 when QoL-AD item 7 is not included in the total score

## Discussion

This work describes the development of a mapping algorithm that can be used to obtain EQ-5D-5L item responses and utilities from observed QoL-AD data. We report mapping algorithms from self-rated QoL-AD to self-rated EQ-5D responses or utilities, and from proxy-rated QoL-AD data to proxy-rated-rated EQ-5D data based on mlogit.

The preferred mapping algorithm, based on prediction performance, uses a response mapping approach based on a multinomial logit model to generate responses to the individual EQ-5D-5L items, and thence utility levels. An advantage of the response mapping approach is that it generates estimates of utility levels for use in cost-effectiveness analyses, as well as the predicted response data for each question. The latter can be used to assess which EQ-5D domains are driving any potential differences or trends observed. This model also allows researchers to attach different country specific value sets if required.

We aimed to generate a mapping algorithm that could be used as widely as possible, requiring as input data only the QoL-AD items (excluding item 7) and the person with dementia’s sex and age. We excluded QoL-AD item 7 responses from our main models, as this question relates to the participant’s marriage. In interviews, unmarried PwD should instead be asked about their closest personal relationship, or their carer, although the question should be classed as missing if there is no one appropriate, or that the PwD are unsure. Our data and the literature [25, 34] indicate that this item tends to be unavailable more often than other data. This approach means that our recommended mapping algorithm can be used even in scenarios where item 7 is unavailable, and does not require the imputation of such data. As such, missing data in the utilities required for cost-effectiveness analyses are minimised, as recommended in the literature [35]. This approach also provided us with a larger dataset and therefore better precision in our results.

We included age and sex in our recommended mapping algorithm, as inclusion of these variables resulted in improved prediction accuracy. As age and sex will commonly be available in studies that seek to implement this mapping algorithm, we do not anticipate problems arising from this added model complexity.

Exploration of the association between observed and predicted EQ-5D indices, and the ranges of EQ-5D indices predicted for given QOL-AD scores, indicate that the models are plausible and intuitive. We observed RMSEs between 0.13 and 0.18, and MAEs between 0.11 and 0.14 in the two main scenarios, in line with corresponding values observed in a review of mapping studies, where RMSEs ranged from 0.084 to 0.20 and MAEs from 0.0011 to 0.19 [36]. Application to an independent validation dataset (proxy-rated QOL-AD mapped to proxy-rated EQ-5D) showed that mapped values of similar, though slightly poorer, prediction accuracy could be achieved, indicating that the mapping algorithm can be validly used in other datasets of similar patient populations.

Prediction accuracy was best for the scenario mapping self-rated QoL-AD to self-rated EQ-5D, possibly indicating higher consistency when both questionnaires are completed by the person with dementia themselves. Scenarios mapping from self-rated QoL-AD to proxy-rated EQ-5D generally had the highest RMSEs likely due to recognised differences between QoL-AD scores based on self-reports and proxy-ratings [25]. The goodness-of-fit statistics indicate that a significant percentage of predicted utilities fall more than 0.1 units away from the observed value. However, it should be borne in mind that the aim of mapping is usually to obtain a mean value and mean difference between groups, not individual prediction, and the overall RMSE statistics are similar to those reported in other mapping studies.

We estimated utilities using the cross-walk to the EQ-5D-3L value set by van Hout et al [31], in line with current NICE recommendations [37]. When a validated EQ-5D-5L value set becomes available for the UK, it should be straightforward to combine that with the response mapping approach reported here.

Our results were derived using a UK value set for the EQ-5D-3L, and the utilities we report may not be valid in other countries where there is reason to think valuations of health states are significantly different. An advantage of the response mapping approach is that different value sets could be applied to the equations we derive, and to that end our code is available to other researchers on request.

While we conducted the mapping study as thoroughly as possible, it is not without limitations. Access to larger datasets might have improved the predictive accuracy of the model, particularly for participants with lower observed QoL-AD and EQ-5D scores, which were not commonly observed in the dataset.

The performance parameters used are within the range of those observed in other mapping studies, although towards the higher end. We found evidence of over-prediction for those with below median observed EQ-5D scores, and under-prediction for those with above median observed EQ-5D scores, a pattern also seen with other mapping studies [32, 38]. Generally, prediction accuracy was worst for participants with lowest QoL-AD scores. For these participants, the largest variation in EQ-5D utilities was observed, and hence outcomes for this subpopulation were more challenging to model. The large amount of variability between the predicted and observed utility values also likely reflects the medium correlation between the QoL-AD and EQ-5D utilities. These patterns were consistent across the estimation and validation datasets. Potential users of the mapping algorithms should be aware that the predicted utility levels will be less reliable as QoL-AD scores decrease.

The prediction accuracy of the mapping algorithms could possibly have been improved by the use of additional explanatory variables. However, additional variables may not always be available in existing datasets, thus limiting the application of a mapping algorithm. In addition, some variables commonly collected in this disease area, including the MMSE and CDR, have been shown to correlate poorly with self-rated QoL scores [39-43], and were therefore not used in the mapping algorithm. We found the mlogit model to be the best-fitting model, although it does not account for the clear ordering of responses of the EQ-5D-5L items. Ologit models take this into account, but assume proportional odds across each category of response, which may not be appropriate in the datasets used.

The validation dataset used the EQ-5D-3L instead of the five-level version. We were thus unable to validate the ability of the model to predict individual responses in an independent dataset.

The recommended mapping algorithm is based on a response mapping model that uses the QoL-AD items. As such, the mapping algorithm can only be used if item-level data are available. The prediction accuracy was reduced when the QoL-AD composite scores were used, and we therefore do not recommend this approach.

While the mlogit model performed best, there were concerns about non-convergence and perfect prediction for some responses, resulting in large and inconsistent regression coefficients for some categories with low observed counts. This may lead to bias being introduced when applying the mapping algorithm in populations dissimilar to the estimation dataset, and we caution against over-interpreting individual coefficients, some of which are non-significant. At a population level, we believe the estimates from the mlogit model provide the most reliable predictions. However, we also provide an option to map QoL-AD data to EQ-5D using the Tobit model. This was chosen over the two-part model, which was estimated on lower numbers and therefore may provide less reliable estimates for item categories with low counts.

Our mapping algorithm currently does not provide estimates around the uncertainty for the predicted EQ-5D utilities, although these may be helpful when basing cost-effectiveness analyses on the mapped EQ-5D utilities. This is in line with other mapping studies. However, we do provide ranges and mean of predicted utility for different observed QoL-AD scores.

We did not use an internal validation set, because our sample size contained insufficient observations in poorer health states to consider splitting the dataset, and partly because of methodological reservations about that approach [35]. These are among the reasons why current guidance on mapping to health utility states does not mandate sampling splitting [44]. The external validation dataset demonstrated consistency in the results of the mapping algorithm for the scenario that mapped proxy-rated QoL-AD to self-rated EQ-5D and proxy-rated QoL-AD to proxy-rated EQ-5D, despite some differences in the patient populations, as discussed in the results section. External validation for the other scenarios would have been beneficial, but no suitable datasets were available to us.

Stata code to apply the mlogit and Tobit mapping algorithms to available QoL-AD data is available in the online supplemental material, and can be used to map all scenarios described in this paper either with or without QoL-AD item 7, depending on the data available. The material also includes the Stata code for the statistical models used, as well as the detailed regression results for the mapping algorithm. We recommend use in existing datasets with available QoL-AD data but no EQ-5D utilities where evaluation of cost-effectiveness is desired. Future research should aim to collect EQ-5D-5L data wherever possible.

## Conclusions

We report here a new mapping algorithm with moderate to good prediction accuracy that allows EQ-5D utilities to be derived from QoL-AD data. This will permit researchers to estimate utilities where QoL-AD are available but no EQ-5D-5L scores have been collected. However, for future research, the collection of the EQ-5D-5L alongside disease-specific measures is recommended wherever possible.

## Electronic supplementary material

Below is the link to the electronic supplementary material.

Supplementary file1 (PDF 4506 kb)

## Abbreviations

CDR: Clinical Dementia Rating
CLAD: Centred least absolute deviation
HRQoL: Health-related quality of life
MAE: Mean absolute error
mlogit: Multinomial logistic regression
MMSE: Mini-mental state examination
ologit: Ordinal logistic regression
OLS: Ordinary least squares
PwD: Person with dementia
RMSE: Root mean square error
QoL: Quality of life
QoL-AD: Quality of Life Alzheimer’s Disease scale

## References

[1] Prince M, Wimo A, Guerchet M, Ali G-C, Wu Y-T, Prina M. World Alzheimer Report 2015. The Global Imapct of Dementia. An Analysis of Prevalence, Incidence, Cost and Trends.: Alzeihmer's Disease International, 2015.

[2] National Institute for Health and Care Excellence. Dementia: assessment, management and support for people living with dementia and their carers. 2018. https://www.nice.org.uk/guidance/ng97 Accessed 29 Aug 2018 30011160

[3] Farina N, Page TE, Daley S (2017). Factors associated with the quality of life of family carers of people with dementia: A systematic review. Alzheimers Dement.

[4] World Health Organisation. WHOQOL: Measuring Quality of Life. 2018. https://www.who.int/healthinfo/survey/whoqol-qualityoflife/en/ Accessed 29 Aug 2018

[5] Schölzel-Dorenbos CJM. Quality of life in dementia: From concept to practice: Radboud University Nijmegen; 2011.

[6] Logsdon RG, Gibbons LE, McCurry SM, Teri L (1999). Quality of life in Alzheimer's disease: Patient and caregiver reports. Journal of Mental Health and Aging.

[7] Smith SC, Lamping DL, Banerjee S (2007). Development of a new measure of health-related quality of life for people with dementia: DEMQOL. Psychol Med.

[8] Brazier J, Dixon S (1995). The use of condition specific outcome measures in economic appraisal. Health Econ.

[9] Kind P (2001). Measuring quality of life in evaluating clinical interventions: An overview. Ann Med.

[10] National Institute for Health and Care Excellence. Guide to the methods of technology appraisal 2013. 2013. https://www.nice.org.uk/process/pmg9/chapter/the-reference-case Accessed 03 Sep 2018 27905712

[11] Rowen D, Mulhern B, Banerjee S (2012). Estimating preference-based single index measures for dementia using DEMQOL and DEMQOL-Proxy. Value Health.

[12] Mulhern B, Rowen D, Brazier J, et al. Development of DEMQOL-U and DEMQOL-PROXY-U: Generation of preference-based indices from DEMQOL and DEMQOL-PROXY for use in economic evaluation. *Health Technol Assess* 2013; **17**(5): v-xv, 1-140. 10.3310/hta17050 PMC4781552 23402232

[13] Bruvik FK, Ulstein ID, Ranhoff AH, Engedal K (2012). The quality of life of people with dementia and their family carers. Dementia and Geriatric Cognitive Disorders.

[14] McCarney R, Fisher P, Iliffe S (2008). Ginkgo biloba for mild to moderate dementia in a community setting: a pragmatic, randomised, parallel-group, double-blind, placebo-controlled trial. International Journal of Geriatric Psychiatry: A journal of the psychiatry of late life and allied sciences.

[15] Meeuwsen EJ, Melis RJ, Van Der Aa GC (2012). Effectiveness of dementia follow-up care by memory clinics or general practitioners: randomised controlled trial. BMJ.

[16] Spector A, Thorgrimsen L, Woods B (2003). Efficacy of an evidence-based cognitive stimulation therapy programme for people with dementia: randomised controlled trial. The British Journal of Psychiatry.

[17] Moniz-Cook E, Vernooij-Dassen M, Woods R (2008). A European consensus on outcome measures for psychosocial intervention research in dementia care. Aging and Mental Health.

[18] Dakin H, Abel L, Burns R, Yang Y (2018). Review and critical appraisal of studies mapping from quality of life or clinical measures to EQ-5D: An online database and application of the MAPS statement. Health Qual Life Outcomes.

[19] Petrou S, Rivero-Arias O, Dakin H (2015). The maps reporting statement for studies mapping onto generic preference-based outcome measures. Value Health.

[20] Gallacher J, de Reydet de Vulpillieres F, Amzal B (2019). Challenges for optimizing real-world evidence in Alzheimer's Disease: The ROADMAP project. Journal of Alzheimers Disease.

[21] Kerpershoek L, de Vugt M, Wolfs C (2016). Access to timely formal dementia care in Europe: Protocol of the Actifcare (ACcess to Timely Formal Care) study. BMC Health Services Research.

[22] Handels RL, Aalten P, Wolfs CA (2012). Diagnostic and economic evaluation of new biomarkers for Alzheimer's disease: the research protocol of a prospective cohort study. BMC Neurology.

[23] Logsdon R, Gibbons LE, McCurry SM, Teri L. Quality of Life in Alzheimer’s Disease (QOL-AD). https://eprovide.mapi-trust.org/instruments/quality-of-life-in-alzheimer-s-disease#languages Accessed 21 Jun 2020.

[24] Dichter MN, Wolschon EM, Meyer G, Kopke S (2016). Cross-cultural adaptation of the German version of the Quality of Life in Alzheimer's Disease scale—Nursing Home version (QoL-AD NH). International Psychogeriatry.

[25] Romhild J, Fleischer S, Meyer G (2018). Inter-rater agreement of the Quality of Life-Alzheimer's Disease (QoL-AD) self-rating and proxy rating scale: secondary analysis of RightTimePlaceCare data. Health Qual Life Outcomes.

[26] Barrios H, Verdelho A, Narciso S, Goncalves-Pereira M, Logsdon R, de Mendonca A (2013). Quality of life in patients with cognitive impairment: validation of the Quality of Life-Alzheimer's Disease scale in Portugal. International Psychogeriatry.

[27] Wolak A, Novella JL, Drame M (2009). Transcultural adaptation and psychometric validation of a French-language version of the QoL-AD. Aging Mental Health.

[28] Stypa V, Haussermann P, Fleiner T, Neumann S. Validity and Reliability of the German Quality of Life-Alzheimer's Disease (QoL-AD) Self-Report Scale. *Journal Alzheimers Disease* 2020. 10.3233/JAD-200400 32675413

[29] Dolan P, Roberts J (2002). Modelling valuations for Eq-5d health states: an alternative model using differences in valuations. Medical Care.

[30] Feng Y, Devlin NJ, Shah KK, Mulhern B, van Hout B (2018). New methods for modelling EQ-5D-5L value sets: An application to English data. Health Economics.

[31] van Hout B, Janssen MF, Feng YS (2012). Interim scoring for the EQ-5D-5L: Mapping the EQ-5D-5L to EQ-5D-3L value sets. Value Health.

[32] Dakin H, Gray A, Murray D (2013). Mapping analyses to estimate EQ-5D utilities and responses based on Oxford Knee Score. Qual Life Res.

[33] Howland M, Allan KC, Carlton CE, Tatsuoka C, Smyth KA, Sajatovic M (2017). Patient-rated versus proxy-rated cognitive and functional measures in older adults. Patient Related Outcome Measures.

[34] Bosboom PR, Alfonso H, Eaton J, Almeida OP (2012). Quality of life in Alzheimer's disease: Different factors associated with complementary ratings by patients and family carers. International Psychogeriatrics.

[35] Leurent B, Gomes M, Carpenter JR (2018). Missing data in trial-based cost-effectiveness analysis: An incomplete journey. Health Economics.

[36] Brazier JE, Yang Y, Tsuchiya A, Rowen DL (2010). A review of studies mapping (or cross walking) non-preference based measures of health to generic preference-based measures. The European Journal of Health Economics.

[37] National Institute for Health and Care Excellence. Position statement on use of the EQ-5D-5L valuation set for England (updated November 2018). 2018. https://www.nice.org.uk/about/what-we-do/our-programmes/nice-guidance/technology-appraisal-guidance/eq-5d-5l Accessed 29 Nov 2018.

[38] Dakin H, Petrou S, Haggard M, Benge S, Williamson I (2010). Mapping analyses to estimate health utilities based on responses to the OM8-30 Otitis Media Questionnaire. Qual Life Res.

[39] Shearer J, Green C, Ritchie CW, Zajicek JP (2012). Health state values for use in the economic evaluation of treatments for Alzheimer’s disease. Drugs & Aging.

[40] Banerjee S, Samsi K, Petrie CD (2009). What do we know about quality of life in dementia? A review of the emerging evidence on the predictive and explanatory value of disease specific measures of health related quality of life in people with dementia. International Journal of Geriatric Psychiatry: A Journal of the Psychiatry of Late Life and Allied Sciences.

[41] Sheehan BD, Lall R, Stinton C (2012). Patient and proxy measurement of quality of life among general hospital in-patients with dementia. Aging Mental Health.

[42] Vogel A, Mortensen EL, Hasselbalch SG, Andersen BB, Waldemar G (2006). Patient versus informant reported quality of life in the earliest phases of Alzheimer's disease. International Journal of Geriatric Psychiatry: A journal of the Psychiatry of Late Life and Allied Sciences.

[43] Lacey L, Bobula J, Rüdell K, Alvir J, Leibman C (2015). Quality of life and utility measurement in a large clinical trial sample of patients with mild to moderate Alzheimer’s disease: determinants and level of changes observed. Value in Health.

[44] Wailoo AJ, Hernandez-Alava M, Manca A (2017). Mapping to estimate health-state utility from non-preference-based outcome measures: An ISPOR good practices for outcomes research task force report. Value Health.

