# Supplementary material for: Obtaining EQ-5D-5L utilities from the disease specific quality of life Alzheimer’s disease scale: development and results from a mapping study
Source: Qual Life Res. 2020 Oct 17;30(3):867–79. doi: 10.1007/s11136-020-02670-8 (PMC7952290; doi:10.1007/s11136-020-02670-8)
Supplement: Supplementary file 1 — Supplementary file1 (PDF 4506 kb) [file 11136_2020_2670_MOESM1_ESM.pdf]

Supplemental material for:

Title

Obtaining EQ-5D-5L utilities from the disease specific Quality of Life Alzheimer's Disease Scale: Development and results from a mapping study – Supplemental material

Journal

Quality of Life Research

Authors

Ines Rombach*, Marvi Iftikhar*, Gurleen S. Jhuti, Anders Gustavsson, Pascal Lecomte, Mark Belger, Ron Handels, Amparo Y. Castro Sanchez, Jan Kors, Louise Hopper, Marcel Olde Rikkert, Geir Selbæk, Astrid Stephan, Sietske A.M. Sikkes, Bob Woods, Manuel Gonçalves-Pereira, Orazio Zanetti, Inez H.G.B. Ramakers, Frans R.J. Verhey, John Gallacher, Actifcare Consortium, LeARN Consortium, Filipa Landeiro*, Alastair M. Gray* on behalf on the ROADMAP Consortium

*Equal contribution as joint first and joint senior authors, respectively

Corresponding Author

Ines Rombach, Health Economics Research Centre, Nuffield Department of Population Health, University of Oxford, Oxford, United Kingdom

E-mail: ines.rombach@ndorms.ox.ac.uk

Table of Contents

Supplemental Table 1: Overview of the data used (continued from main text) 3

Supplemental Table 2: Item responses to the EQ-5D-5L and QoL-AD 4

Supplemental Table 3: Characteristics of participants whose observations were included in and excluded from mapping study 7

Supplemental Figure 1: Scatter plots of observed QoL-AD vs. observed EQ-5D utilities 8

Supplemental Table 4: Correlations between the QoL-AD and EQ-5D-5L (EQ-5D-3L for the validation dataset) 9

Supplemental Table 5a: Comparison of the main mapping algorithms (all scenarios) 11

Supplemental Table 5b: Comparison of the mapping algorithms (excluding QoL-AD question 7, not including age and sex)... 12

Supplemental Table 5c: Comparison of the mapping algorithms (including QoL-AD item 7, age and sex).. 13

Supplemental Table 5d: Comparison of the mapping algorithms when the model selection is run in the validation dataset (excluding QoL-AD item 7, age and sex) 14

Supplemental Figure 2a: Prediction accuracy of the mapping models using mlogit (continued from main manuscript) 15

Supplemental Figure 2b: Prediction accuracy of the mapping models using Tobit......................... 16

Supplemental Figure 2c: Prediction accuracy of the mapping models using Tobit 17

Supplemental Table 6: Assessment of preferred mlogit model across different centiles of QoL-AD scores 18

Supplemental Figure 3: Probability of predicting each response level for a given observed response to the EQ-5D-5L items.. 19

Supplemental Table 7: Mean and range of predicted EQ-5D utilities for observed QoL-AD scores ... 31

Supplemental Figure 4: Range of predicted EQ-5D utilities for observed QoL-AD scores................. 32

Instructions for the Stata ado-file to map the QoL-AD to the EQ-5D-5L 33

Supplemental Table 1: Overview of the data used (continued from main text)

	Estimation datasets		Validation dataset
**Demographic Variable**	**Proxy-rated QoL-AD → Self-rated EQ-5D**	**Self-rated QoL-AD → Proxy-rated EQ-5D**	**Proxy-rated QoL-AD → Self- rated EQ-5D**
Total number of observations in datasets	1,353	1,353	753
Total number of observations excluded from analysis*	334 (25%)	336 (25%)	366 (49%)
Total number of observations included in analysis	1,019 (75%)	1,017 (75%)	387 (51%)
Total number of observations excluded if QoL-AD item 7 was included in analysis	*149/1,019(15%)*	*139/1,017 (14%)*	46/387 (12%)
Number of participants included in analysis	429	427	204
PwD Age (SD)	78 (8)	78 (8)	67 (9)
Proxy Age (SD)	66 (13)	66 (13)	62 (11)
PwD Sex (Female)	55%	55%	32%
Proxy Sex (Female)	67%	67%	76%
MMSE**	19 (5)	19 (5)	26 (4)
CDR 0***	<1%	0%	12%
CDR 0.5***	3%	3%	54%
CDR 1***	70%	69%	30%
CDR 2***	26%	26%	4%
CDR 3***	1%	1%	0%
Self-rated QoL-AD mean (SD)	n/a	35 (6)	n/a
Self-rated QoL-AD median (range)	n/a	36 (16, 52)	n/a
Proxy-rated QoL-AD mean (SD)	30 (6)	n/a	32 (5)
Proxy-rated QoL-AD median (range)	30 (15, 50)	n/a	31 (15, 51)
Self-rated EQ-5D Utility mean (SD)	0.77 (0.21)	n/a	0.83 (0.20)
Self-rated EQ-5D Utility median (range)	0.81 (-0.26, 1)	n/a	0.85 (-0.04, 1)
Proxy-rated EQ-5D Utility mean (SD)	n/a	0.62 (0.23)	n/a
Proxy-rated EQ-5D Utility median (range)	n/a	0.65 (-0.31, 1)	n/a
Spearman's Correlation (95% CI)	0.32 (0.27, 0.38)	0.24 (0.18, 0.30)	0.21 (0.11, 0.30)

**Insufficient EQ-5D-5L or QOL-AD data were available for inclusion in the mapping study, either through unavailability of the complete questionnaire, or individual items.*
***MMSE (Mini-mental state examination) data were unavailable for a proportion of people with dementia. the following percentage of the total number of observations are excluded from the MMSE summaries: Estimation dataset: 8% in both the 'Self-rated QoL-AD → Self-rated EQ-5D' scenario and the 'Proxy-rated QoL-AD → Proxy- rated EQ-5D' scenario; Validation dataset: 1%. The score ranges from 0 to 30, with higher scores indicating less cognitive impairment.*

****CDR (clinical dementia rating) data were unavailable for a proportion of observations; the following percentage of the total number of observations are excluded from the CRD summaries: estimation dataset: 0.5% in the 'Self-rated QoL-AD → Proxy-rated EQ-5D' scenario; 2% in the 'Proxy-rated QoL-AD → Self- rated EQ-5D' scenario ; validation dataset: 7%. Missing data occurred predominantly due to CDR assessments not being performed, rather than individual domains of cognitive and functional performance being missing. The percentages presented are based on the population with available CDR data only.*

Abbreviations: CI – Confidence interval; PwD – Person with dementia; SD – Standard deviation

Supplemental Table 2: Item responses to the EQ-5D-5L and QoL-AD

Note: the datasets used are the estimation datasets presented in Table 1 in the main text.

	**Self-rated QoL-AD** → **Self-rated EQ-5D** (N=1020)	**Proxy-rated QoL-AD** → **Proxy- rated EQ-5D** (N=1099)
**Item responses to the EQ-5D**		
Mobility		
I have no problems in walking about	582 (57%)	441 (40%)
I have slight problems in walking about	207 (20%)	265 (24%)
I have moderate problems in walking about	174 (17%)	246 (22%)
I have severe problems in walking about	50 (5%)	124 (11%)
I am unable to walk about	7 (1%)	23 (2%)
Self-care		
I have no problems washing or dressing myself	773 (76%)	494 (45%)
I have slight problems washing or dressing myself	142 (14%)	290 (26%)
I have moderate problems washing or dressing myself	68 (7%)	189 (17%)
I have severe problems washing or dressing myself	19 (2%)	67 (6%)
I am unable to wash or dress myself	18 (2%)	59 (5%)
Usual activities (e.g. work, study, housework, family or leisure activities)		
I have no problems doing my usual activities	590 (58%)	233 (21%)
I have slight problems doing my usual activities	239 (23%)	279 (25%)
I have moderate problems doing my usual activities	125 (12%)	295 (27%)
I have severe problems doing my usual activities	47 (5%)	196 (18%)
I am unable to do my usual activities	19 (2%)	96 (9%)
Pain/discomfort		
I have no pain or discomfort	583 (57%)	418 (38%)
I have slight pain or discomfort	232 (23%)	328 (30%)
I have moderate pain or discomfort	162 (16%)	285 (26%)
I have severe pain or discomfort	36 (4%)	64 (6%)
I have extreme pain or discomfort	7 (1%)	4 (0%)
Anxiety/depression		
I am not anxious or depressed	611 (60%)	404 (37%)
I am slightly anxious or depressed	272 (27%)	349 (32%)
I am moderately anxious or depressed	115 (11%)	272 (25%)
I am severely anxious or depressed	19 (2%)	64 (6%)
I am extremely anxious or depressed	3 (0%)	10 (1%)
**Item response to the QoL-AD**		
1. How do you feel about your physical health?		
Poor	76 (7%)	189 (17%)
Fair	307 (30%)	393 (36%)

	**Self-rated QoL-AD → Self-rated EQ-5D** (N=1020)	**Proxy-rated QoL-AD → Proxy- rated EQ-5D** (N=1099)
Good	523 (51%)	435 (40%)
Excellent	114 (11%)	82 (7%)
2. How do you feel about your energy level?		
Poor	124 (12%)	362 (33%)
Fair	336 (33%)	422 (38%)
Good	466 (46%)	274 (25%)
Excellent	94 (9%)	41 (4%)
3. How has your mood been lately?		
Poor	76 (7%)	125 (11%)
Fair	274 (27%)	486 (44%)
Good	595 (58%)	439 (40%)
Excellent	75 (7%)	49 (4%)
4. How about your living situation? How do you feel about the place you live now?		
Poor	15 (1%)	68 (6%)
Fair	115 (11%)	168 (15%)
Good	572 (56%)	590 (54%)
Excellent	318 (31%)	273 (25%)
5. How about your memory?		
Poor	262 (26%)	686 (62%)
Fair	486 (48%)	341 (31%)
Good	257 (25%)	64 (6%)
Excellent	15 (1%)	8 (1%)
6. How about your family and your relationship with family members?		
Poor	14 (1%)	44 (4%)
Fair	88 (9%)	178 (16%)
Good	575 (56%)	616 (56%)
Excellent	343 (34%)	261 (24%)
7. How do you feel about your marriage? How is your relationship with (spouse's name)?		
Poor	18 (2%)	39 (4%)
Fair	64 (6%)	150 (14%)
Good	448 (44%)	526 (48%)
Excellent	353 (35%)	228 (21%)
Missing data	137 (13%)	156 (14%)
8. How would you describe your current relationship with your friends?		
Poor	102 (10%)	236 (21%)
Fair	235 (23%)	283 (26%)
Good	548 (54%)	487 (44%)
Excellent	135 (13%)	93 (8%)

	**Self-rated QoL-AD → Self-rated EQ-5D** (N=1020)	**Proxy-rated QoL-AD → Proxy- rated EQ-5D** (N=1099)
9. How do you feel about yourself - when you think of your whole self, and all the different things about you?		
Poor	63 (6%)	139 (13%)
Fair	326 (32%)	432 (39%)
Good	554 (54%)	482 (44%)
Excellent	77 (8%)	46 (4%)
10. How do you feel about your ability to do things like chores around the house or other things you need to do?		
Poor	147 (14%)	504 (46%)
Fair	315 (31%)	342 (31%)
Good	474 (46%)	215 (20%)
Excellent	84 (8%)	38 (3%)
11. How about your ability to do things for fun, that you enjoy?		
Poor	137 (13%)	341 (31%)
Fair	304 (30%)	366 (33%)
Good	483 (47%)	344 (31%)
Excellent	96 (9%)	48 (4%)
12. How do you feel about your current situation with money, your financial situ		
Poor	57 (6%)	213 (19%)
Fair	220 (22%)	258 (23%)
Good	626 (61%)	495 (45%)
Excellent	117 (11%)	133 (12%)
13. How would you describe your life as a whole?		
Poor	34 (3%)	85 (8%)
Fair	249 (24%)	428 (39%)
Good	580 (57%)	530 (48%)
Excellent	157 (15%)	56 (5%)

Supplemental Table 3: Characteristics of participants whose observations were included in and excluded from mapping study

Demographic Variable	Self-rated QoL-AD → Self-rated EQ-5D		Proxy-rated QoL-AD → Proxy-rated EQ-5D		Proxy-rated QoL-AD → Self-rated EQ-5D		Self-rated QoL-AD → Proxy-rated EQ-5D	
**Mapping study**	**Included**	**Excluded***	**Included**	**Excluded***	**Included**	**Excluded***	**Included**	**Excluded***
N	1,020	333	1,099	254	1,019	334	1,017	336
PwD Age (SD)	78 (8)	80 (8)	78 (8)	80 (8)	78 (8)	80 (8)	78 (8)	80 (8)
Proxy Age (SD)	66 (13)	69 (14)	67 (13)	68 (13)	66 (13)	68 (13)	66 (13)	68 (14)
PwD Sex (Female)	55%	53%	54%	56%	55%	52%	55%	535
Proxy Sex (Female)	67%	66%	67%	69%	67%	67%	67%	68%
MMSE	19 (5)	17 (7)	19 (5)	17 (6)	19 (5)	17 (7)	19 (5)	17 (7)
CDR 0	0%	1%	0%	1%	<1%	0%	0%	1%
CDR 0.5	3%	2%	3%	3%	3%	2%	3%	2%
CDR 1	70%	46%	67%	60%	70%	46%	69%	48%
CDR 2	26%	39%	28%	32%	26%	38%	26%	37%
CDR 3	1%	12%	3%	4%	1%	14%	1%	12%
Self-rated QoL-AD mean (SD)	35 (6)	34 (10)	n/a	n/a	n/a	n/a	35 (6)	33 (9)
Self-rated QoL-AD median (range)	36 (16, 52)	35 (13, 48)	n/a	n/a	n/a	n/a	36 (16, 52)	36 (13, 49)
Proxy-rated QoL-AD mean (SD)	n/a	n/a	30 (6)	32 (3)	30 (6)	29 (5)	n/a	n/a
Proxy-rated QoL-AD median (range)	n/a	n/a	30 (15, 50)	34 (27, 36)	30 (15, 50)	29 (18, 41)	n/a	n/a
Self-rated EQ-5D Utility mean (SD)	0.77 (0.21)	0.76 (0.20)	n/a	n/a	0.77 (0.21)	0.73 (0.22)	n/a	n/a
Self-rated EQ-5D Utility median (range)	0.81 (-0.26, 1)	0.77 (0.02, 1)	n/a	n/a	0.81 (-0.26, 1)	0.76 (-0.15, 1)	n/a	n/a
Proxy-rated EQ-5D Utility mean (SD)	n/a	n/a	0.60 (0.24)	0.53 (0.25)	n/a	n/a	0.62 (0.23)	0.44 (0.28)
Proxy-rated EQ-5D Utility median (range)	n/a	n/a	0.64(-0.31, 1)	0.59 (-0.21, 1)	n/a	n/a	0.65 (-0.31, 1)	0.50 (-0.22, 1)

**participants were excluded from the mapping study if either the EQ-5D Utility, the QoL-AD or both were missing; hence the summaries for these variables only include a subset of the sample.*

Abbreviations: CI – Confidence interval; PwD – Person with dementia; SD – Standard deviation

Supplemental Figure 1: Scatter plots of observed QoL-AD vs. observed EQ-5D utilities

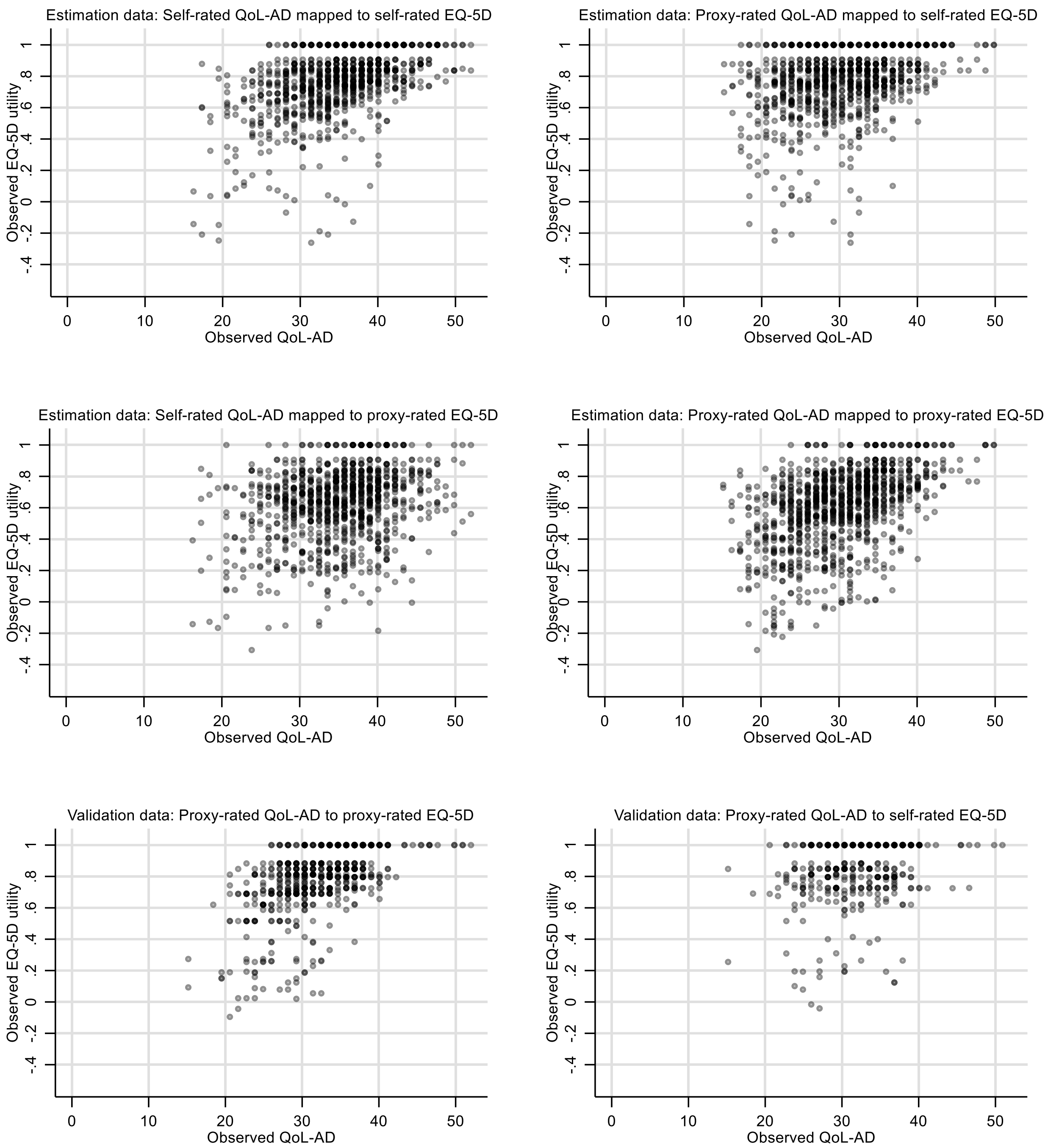

Supplemental Table 4: Correlations between the QoL-AD and EQ-5D-5L (EQ-5D-3L for the validation dataset)

			Qol-AD item 1 (Physical health)	Qol-AD item 2 (Energy levels)	Qol-AD item 3 (Mood)	Qol-AD item 4 (Living situation)	Qol-AD item 5 (Memory)	Qol-AD item 6 (Family)	Qol-AD item 7* (Marriage)	Qol-AD item 8 (Friends)	Qol-AD item 9 (Self)	Qol-AD item 10 (Chores)	Qol-AD item 11 (Fun)	Qol-AD item 12 (Money)	Qol-AD item 13 (Life as a whole)
Estimation datasets: Actifcare	Self-rated QoL-AD vs. self-rated EQ-5D	Mobility	-0.39	-0.39	-0.25	-0.16	-0.22	-0.11	-0.15	-0.15	-0.27	-0.38	-0.26	-0.17	-0.21
		Self-care	-0.28	-0.28	-0.15	-0.14	-0.09	-0.15	-0.12	-0.17	-0.17	-0.39	-0.22	-0.19	-0.23
		Usual activities	-0.33	-0.39	-0.25	-0.23	-0.21	-0.15	-0.15	-0.22	-0.27	-0.53	-0.31	-0.17	-0.29
		Pain	-0.33	-0.29	-0.24	-0.18	-0.18	-0.04	-0.09	-0.02	-0.27	-0.19	-0.18	-0.08	-0.23
		Anxiety/depression	-0.28	-0.31	-0.44	-0.22	-0.23	-0.12	-0.12	-0.16	-0.37	-0.24	-0.22	-0.15	-0.32
	Proxy-rated QoL-AD vs. Self-rated EQ-5D	Mobility	-0.43	-0.28	-0.15	-0.11	0.03	-0.02	-0.07	-0.12	-0.18	-0.26	-0.19	-0.06	-0.15
		Self-care	-0.26	-0.18	-0.09	-0.12	-0.03	-0.06	-0.09	-0.05	-0.15	-0.24	-0.19	-0.09	-0.13
		Usual activities	-0.34	-0.23	-0.14	-0.13	-0.05	-0.04	-0.04	-0.11	-0.22	-0.27	-0.18	-0.10	-0.15
		Pain	-0.28	-0.16	-0.13	-0.13	0.02	0.05	-0.07	-0.03	-0.16	-0.06	-0.05	-0.05	-0.11
		Anxiety/depression	-0.21	-0.12	-0.26	-0.13	-0.03	-0.06	-0.08	-0.05	-0.24	-0.03	-0.14	-0.08	-0.19
	Self-rated QoL-AD vs. Proxy-rated EQ-5D	Mobility	-0.26	-0.31	-0.10	-0.06	-0.06	-0.10	-0.07	-0.10	-0.15	-0.34	-0.19	-0.10	-0.12
		Self-care	-0.16	-0.19	-0.07	-0.07	0.02	-0.09	-0.06	-0.12	-0.11	-0.32	-0.19	-0.16	-0.12
		Usual activities	-0.11	-0.21	-0.03	0.02	0.01	-0.01	0.00	-0.03	-0.05	-0.28	-0.14	-0.02	-0.05
		Pain	-0.21	-0.22	-0.14	-0.12	-0.10	-0.04	-0.06	-0.05	-0.17	-0.17	-0.12	-0.08	-0.20
		Anxiety/depression	-0.14	-0.13	-0.18	-0.09	-0.05	-0.04	-0.05	-0.07	-0.18	-0.08	-0.14	-0.06	-0.20
	Proxy-rated QoL-AD vs. Proxy-rated EQ-5D	Mobility	-0.47	-0.35	-0.11	-0.09	-0.04	-0.09	-0.04	-0.15	-0.12	-0.36	-0.24	-0.08	-0.12
		Self-care	-0.25	-0.25	-0.15	-0.17	-0.11	-0.15	-0.15	-0.19	-0.19	-0.47	-0.29	-0.14	-0.22
		Usual activities	-0.30	-0.33	-0.16	-0.05	-0.18	-0.06	-0.08	-0.19	-0.19	-0.52	-0.26	-0.11	-0.23
		Pain	-0.39	-0.25	-0.21	-0.13	-0.06	0.02	-0.06	-0.06	-0.18	-0.14	-0.11	-0.08	-0.12
		Anxiety/depression	-0.20	-0.23	-0.38	-0.14	-0.15	-0.08	-0.10	-0.14	-0.33	-0.11	-0.21	-0.13	-0.30
Validation dataset	Proxy-rated QoL-AD vs. Self-rated EQ-5D	Mobility	-0.34	-0.26	-0.05	-0.08	-0.04	0.03	-0.02	-0.07	-0.08	-0.16	-0.22	-0.07	-0.07
		Self-care	-0.16	-0.11	-0.07	0.00	0.07	-0.02	-0.07	0.00	-0.12	-0.21	-0.16	0.09	-0.11
		Usual activities	-0.24	-0.21	-0.13	-0.03	-0.01	-0.09	-0.09	-0.09	-0.15	-0.16	-0.23	0.01	-0.21
		Pain	-0.33	-0.15	0.02	-0.07	-0.03	0.03	0.04	0.03	0.00	0.02	-0.06	-0.05	-0.03
		Anxiety/depression	-0.18	-0.13	-0.24	-0.03	-0.05	-0.14	-0.13	-0.20	-0.18	0.03	-0.12	-0.02	-0.18

**Validation dataset**	**Proxy- rated QoL-AD vs. Proxy-rated EQ-5D**	Mobility	-0.39	-0.31	-0.10	-0.11	-0.09	-0.05	0.01	-0.12	-0.20	-0.29	-0.30	-0.12	-0.20
		Self-care	-0.29	-0.29	-0.14	-0.11	-0.08	-0.10	-0.09	-0.21	-0.27	-0.43	-0.35	-0.16	-0.30
		Usual activities	-0.31	-0.44	-0.30	-0.16	-0.27	-0.14	-0.16	-0.24	-0.41	-0.56	-0.42	-0.23	-0.43
		Pain	-0.51	-0.33	-0.18	-0.06	-0.10	-0.02	-0.05	-0.13	-0.20	-0.17	-0.25	-0.09	-0.23
		Anxiety/depression	-0.19	-0.25	-0.58	-0.17	-0.23	-0.19	-0.27	-0.22	-0.40	-0.18	-0.30	-0.19	-0.42

Cells highlighted in green indicate strong correlations (absolute value of 0.30 or higher), and cells highlighted in red indicated correlations close to 0 (absolute value 0 to 0.10)

Supplemental Table 5a: Comparison of the main mapping algorithms (all scenarios)

Model	Self-rated QoL-AD → Self-rated EQ-5D					Proxy-rated QoL-AD → Self-rated EQ-5D				
	RMSE	MAE	Minimum predicted score[1]	Maximum predicted score[1]	Accuracy within 0.1 points	RMSE	MAE	Minimum predicted score[1]	Maximum predicted score[1]	Accuracy within 0.1 points
**Direct OLS Continuous**	0.1797	0.1302	0.460	1.000	49%	0.1937	0.1413	0.607	1.000	47%
**Direct OLS Categorical**	0.1614	0.1196	0.249	1.000	51%	0.1809	0.1297	0.494	1.000	50%
**Direct Tobit**	0.1612	0.1191	0.233	0.974	52%	0.1805	0.1291	0.484	0.989	50%
**Direct Clad**	0.1677	0.1195	0.293	1.000	54%	0.1947	0.1322	0.468	1.000	51%
**Direct 2-part**	0.1610	0.1192	0.259	0.982	52%	0.1802	0.1291	0.485	0.995	50%
**Response OLS Categorical**	0.1765	0.1267	0.378	1.000	49%	0.1810	0.1308	0.548	1.000	44%
**Response OLS Continuous**	0.1913	0.1386	0.533	1.000	45%	0.1986	0.1461	0.573	1.000	41%
**Response ologit**	0.1624	0.1196	0.210	0.964	51%	0.2015	0.1495	0.429	0.975	50%
**Response mlogit**	**0.1348**	**0.1063**	-0.253	0.972	53%	**0.1580**	**0.1207**	-0.165	0.976	50%

Model	Self-rated QoL-AD → Proxy-rated EQ-5D					Proxy-rated QoL-AD → Proxy-rated EQ-5D				
	RMSE	MAE	Minimum predicted score[2]	Maximum predicted score[2]	Accuracy within 0.1 points	RMSE	MAE	Minimum predicted score[2]	Maximum predicted score[2]	Accuracy within 0.1 points
**Direct OLS Continuous**	0.2159	0.1686	0.397	0.844	38%	0.2109	0.1614	0.336	1.000	42%
**Direct OLS Categorical**	0.2008	0.1563	0.224	0.912	41%	0.1916	0.1473	0.233	1.000	44%
**Direct Tobit**	0.2007	0.1562	0.217	0.882	42%	0.1915	0.1471	0.229	0.958	45%
**Direct Clad**	0.2082	0.1563	0.227	1.000	43%	0.2012	0.1497	0.200	1.000	45%
**Direct 2-part**	0.2007	0.1563	0.232	0.907	41%	0.1913	0.1465	0.241	0.986	45%
**Response OLS Categorical**	0.2111	0.1666	0.377	1.000	38%	0.2062	0.1555	0.378	1.000	42%
**Response OLS Continuous**	0.2171	0.1705	0.325	0.906	37%	0.2140	0.1633	0.393	1.000	40%
**Response ologit**	0.2018	0.1583	0.216	0.857	39%	0.1928	0.1491	0.172	0.921	43%
**Response mlogit**	**0.1899**	**0.1503**	-0.076	0.840	43%	**0.1819**	**0.1401**	-0.051	0.930	47%

The lowest root mean square error (RMSE) and mean absolute error (MAE) are highlighted in bold

[1]*The observed minimum and maximum observed self-rated EQ-5D scores were -0.261 and 1, respectively*

[2]*The observed minimum and maximum observed proxy-rated EQ-5D scores were -0.307 and 1, respectively*

Supplemental Table 5b: Comparison of the mapping algorithms (excluding QoL-AD question 7, not including age and sex)

Model	Self-rated QoL-AD → Self-rated EQ-5D					Proxy-rated QoL-AD → Self-rated EQ-5D				
	RMSE	MAE	Minimum predicted score[1]	Maximum predicted score[1]	Accuracy within 0.1 points	RMSE	MAE	Minimum predicted score[1]	Maximum predicted score[1]	Accuracy within 0.1 points
Direct OLS Continuous	0.1812	0.1316	0.460	1.000	48%	0.1956	0.1428	0.609	0.988	46%
Direct OLS Categorical	0.1625	0.1209	0.266	0.987	50%	0.1828	0.1314	0.488	1.000	49%
Direct Tobit	0.1622	0.1203	0.251	0.961	51%	0.1827	0.1309	0.481	0.981	50%
Direct Clad	0.1682	0.1206	0.197	1.000	52%	0.1956	0.1337	0.470	1.000	50%
Direct 2-part	0.1623	0.1208	0.274	0.969	50%	0.1827	0.1312	0.486	0.983	50%
Response OLS Categorical	0.1801	0.1309	0.409	1.000	46%	0.1992	0.1480	0.548	1.000	43%
Response OLS Continuous	0.1945	0.1426	0.533	1.000	42%	0.2017	0.1515	0.573	1.000	40%
Response ologit	0.1635	0.1208	0.229	0.950	50%	0.1834	0.1326	0.429	0.962	49%
Response mlogit	**0.1410**	**0.1103**	-0.219	0.963	51%	**0.1613**	**0.1233**	-0.144	0.964	49%
	**Self-rated QoL-AD → Proxy-rated EQ-5D**					**Proxy-rated QoL-AD → Proxy-rated EQ-5D**				
	RMSE	MAE	Minimum predicted score[2]	Maximum predicted score[2]	Accuracy within 0.1 points	RMSE	MAE	Minimum predicted score[2]	Maximum predicted score[2]	Accuracy within 0.1 points
Direct OLS Continuous	0.2192	0.1711	0.441	0.788	38%	0.2145	0.1633	0.324	0.968	40%
Direct OLS Categorical	0.2038	0.1591	0.245	0.888	40%	0.1939	0.1487	0.241	0.988	44%
Direct Tobit	0.2037	0.1589	0.241	0.862	40%	0.1939	0.1486	0.238	0.945	44%
Direct Clad	0.2115	0.1588	0.295	0.983	43%	0.2026	0.1524	0.224	1.000	43%
Direct 2-part	0.2035	0.1592	0.256	0.883	40%	0.1935	0.1477	0.247	0.987	44%
Response OLS Categorical	0.2140	0.1692	0.377	1.000	37%	0.2081	0.1573	0.378	1.000	41%
Response OLS Continuous	0.2201	0.1742	0.409	0.906	35%	0.2154	0.1650	0.393	1.000	39%
Response ologit	0.2049	0.1604	0.236	0.847	39%	0.1955	0.1506	0.193	0.929	44%
Response mlogit	**0.1941**	**0.1535**	-0.023	0.857	41%	**0.1862**	**0.1423**	-0.047	0.945	47%

The lowest root mean square error (RMSE) and mean absolute error (MAE) are highlighted in bold
[1]The observed minimum and maximum self-rated EQ-5D scores were -0.261 and 1, respectively
[2]The observed minimum and maximum proxy-rated EQ-5D scores were -0.307 and 1, respectively

Supplemental Table 5c: Comparison of the mapping algorithms (including QoL-AD item 7, age and sex)

Model	Self-rated QoL-AD → Self-rated EQ-5D					Proxy-rated QoL-AD → Self-rated EQ-5D				
	RMSE	MAE	Minimum predicted score[1]	Maximum predicted score[1]	Accuracy within 0.1 points	RMSE	MAE	Minimum predicted score[1]	Maximum predicted score[1]	Accuracy within 0.1 points
Direct OLS Continuous	0.1835	0.1326	0.459	1.000	47%	0.1948	0.1435	0.603	1.000	45%
Direct OLS Categorical	0.1625	0.1197	0.263	1.000	51%	0.1800	0.1301	0.447	1.000	49%
Direct Tobit	0.1620	0.1191	0.241	0.982	51%	0.1794	0.1295	0.426	0.993	50%
Direct Clad	0.1714	0.1210	0.190	1.000	53%	0.1934	0.1328	0.533	1.000	50%
Direct 2-part	0.1627	0.1195	0.266	0.989	52%	0.1790	0.1296	0.438	1.000	49%
Response OLS Categorical	0.1815	0.1299	0.333	1.000	47%	0.1979	0.1449	0.533	1.000	44%
Response OLS Continuous	0.1928	0.1400	0.533	1.000	45%	0.1996	0.1474	0.567	1.000	43%
Response ologit	0.1636	0.1192	0.184	0.975	52%	0.1800	0.1307	0.352	0.984	49%
Response mlogit	**0.1335**	**0.1050**	-0.259	0.981	54%	**0.1569**	**0.1194**	-0.179	0.983	50%

Model	Self-rated QoL-AD → Proxy-rated EQ-5D					Proxy-rated QoL-AD → Proxy-rated EQ-5D				
	RMSE	MAE	Minimum predicted score[2]	Maximum predicted score[2]	Accuracy within 0.1 points	RMSE	MAE	Minimum predicted score[2]	Maximum predicted score[2]	Accuracy within 0.1 points
Direct OLS Continuous	0.2190	0.1711	0.418	0.843	37%	0.2135	0.1634	0.336	1.000	41%
Direct OLS Categorical	0.2011	0.1573	0.218	0.927	40%	0.1918	0.1475	0.266	1.000	44%
Direct Tobit	0.2009	0.1572	0.206	0.891	40%	0.1917	0.1472	0.255	0.967	45%
Direct Clad	0.2094	0.1591	0.185	1.000	41%	0.2004	0.1461	0.238	1.000	47%
Direct 2-part	0.2006	0.1570	0.235	0.932	40%	0.1910	0.1462	0.266	0.994	45%
Response OLS Categorical	0.2123	0.1681	0.350	1.000	38%	0.2062	0.1548	0.211	1.000	42%
Response OLS Continuous	0.2181	0.1711	0.409	0.906	38%	0.2147	0.1644	0.393	1.000	40%
Response ologit	0.2020	0.1591	0.159	0.862	39%	0.1936	0.1497	0.181	0.937	44%
Response mlogit	**0.1883**	**0.1493**	-0.107	0.856	43%	**0.1785**	**0.1372**	-0.196	0.950	48%

The lowest root mean square error (RMSE) and mean absolute error (MAE) are highlighted in bold

[1]The observed minimum and maximum self-rated EQ-5D scores were -0.261 and 1, respectively

[2]The observed minimum and maximum self-rated EQ-5D scores were -0.307 and 1, respectively

Supplemental Table 5d: Comparison of the mapping algorithms when the model selection is run in the validation dataset (excluding QoL-AD item 7, age and sex)

Model	Proxy-rated QoL-AD → Self-rated EQ-5D					Proxy- rated QoL-AD → Proxy- rated EQ-5D				
	RMSE	MAE	Minimum predicted score[1]	Maximum predicted score[1]	Accuracy within 0.1 points	RMSE	MAE	Minimum predicted score[2]	Maximum predicted score[2]	Accuracy within 0.1 points
**Direct OLS Continuous**	0.1970	0.1441	0.690	1.000	42%	0.1774	0.1275	0.429	1.000	52%
**Direct OLS Categorical**	0.1741	0.1276	0.342	1.000	51%	0.1565	0.1117	0.191	1.000	59%
**Direct Tobit**	0.1743	0.1257	0.319	0.999	53%	0.1571	0.1132	0.184	0.998	59%
**Direct Clad**	n/a	n/a	n/a	n/a	n/a	n/a	n/a	n/a	n/a	n/a
**Direct 2-part**	0.1747	0.1260	0.298	1.000	52%	0.1556	0.1108	0.224	1.000	59%
**Response OLS Categorical**	0.1986	0.1281	0.587	1.000	48%	0.1722	0.1070	0.024	1.000	58%
**Response OLS Continuous**	0.2095	0.1325	0.620	1.000	49%	0.1792	0.1118	0.516	1.000	58%
**Response ologit**	0.1729	0.1244	0.193	0.999	53%	0.1556	0.1102	0.126	1.000	61%
**Response mlogit**	**0.1428**	**0.1061**	0.056	0.999	59%	**0.1391**	**0.0994**	-0.105	1.000	65%

The lowest root mean square error (RMSE) and mean absolute error (MAE) are highlighted in bold. The direct CLAD model was not performed for these smaller sample sizes due to convergence issues.
[1]*The observed minimum and maximum self-rated EQ-5D scores were -0.041 and 1, respectively*
[2]*The observed minimum and maximum proxy-rated EQ-5D scores were -0.095 and 1, respectively*

When the mapping algorithm derived from the estimation dataset is applied to the validation dataset to estimate self-rated EQ-5D utilities from proxy-rated QoL-AD data, a RMSE of 0.2152 and a MAE of 0.1542 are obtained.

Supplemental Figure 2a: Prediction accuracy of the mapping models using mlogit (continued from main manuscript)

For all plots: Scatter plots of predicted versus observed utilities are presented in the left-hand column. Darker markers on the graphs indicate overlapping data points. Observed utilities have been classed into quartiles in the right-hand column, and the means of these quartiles are shown on the x-axis. On the y-axis, the median, interquartile range (thicker, darker vertical lines) and 10th to 90th centiles (thinner, lighter vertical lines) of the predicted utilities are shown on the y-axis to represent the data distribution.

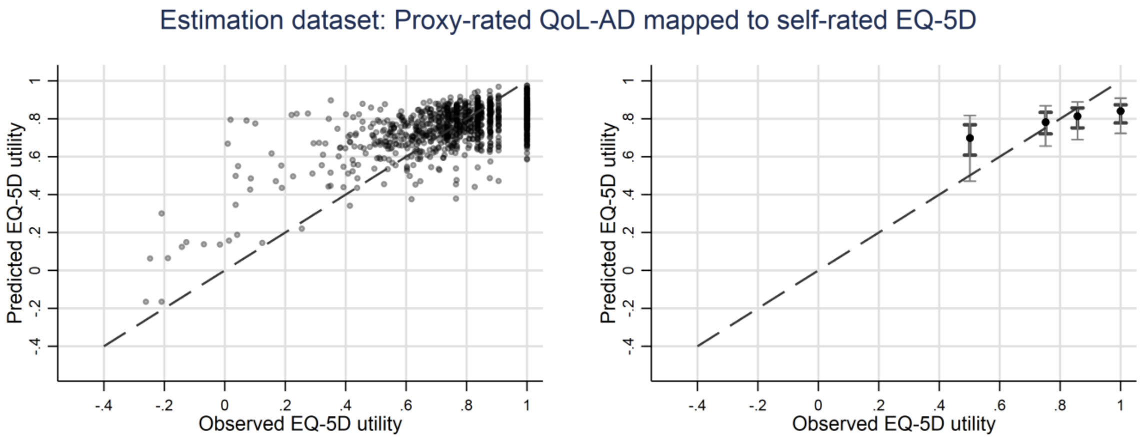

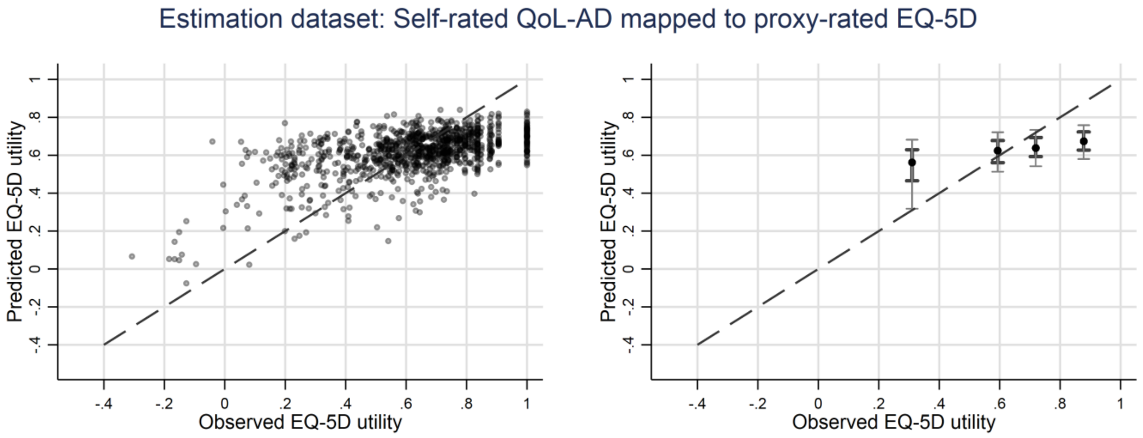

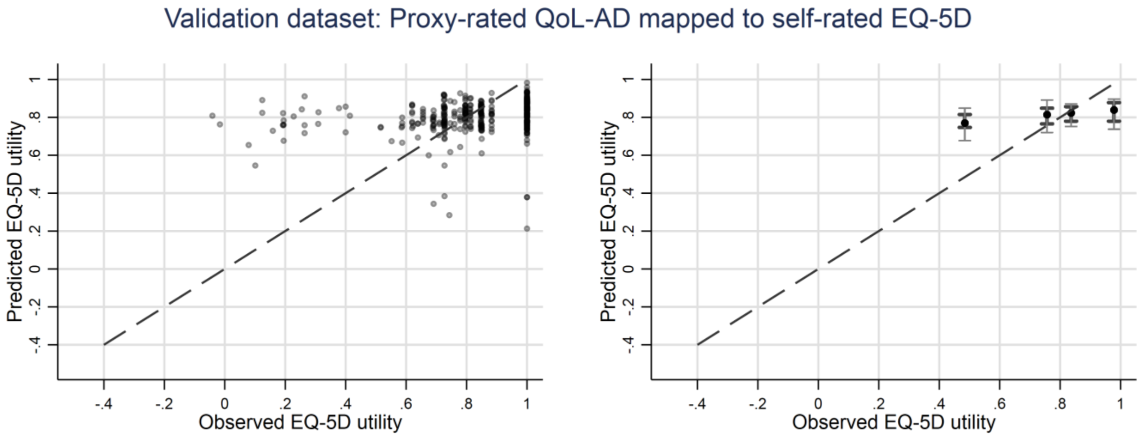

Supplemental Figure 2b: Prediction accuracy of the mapping models using Tobit

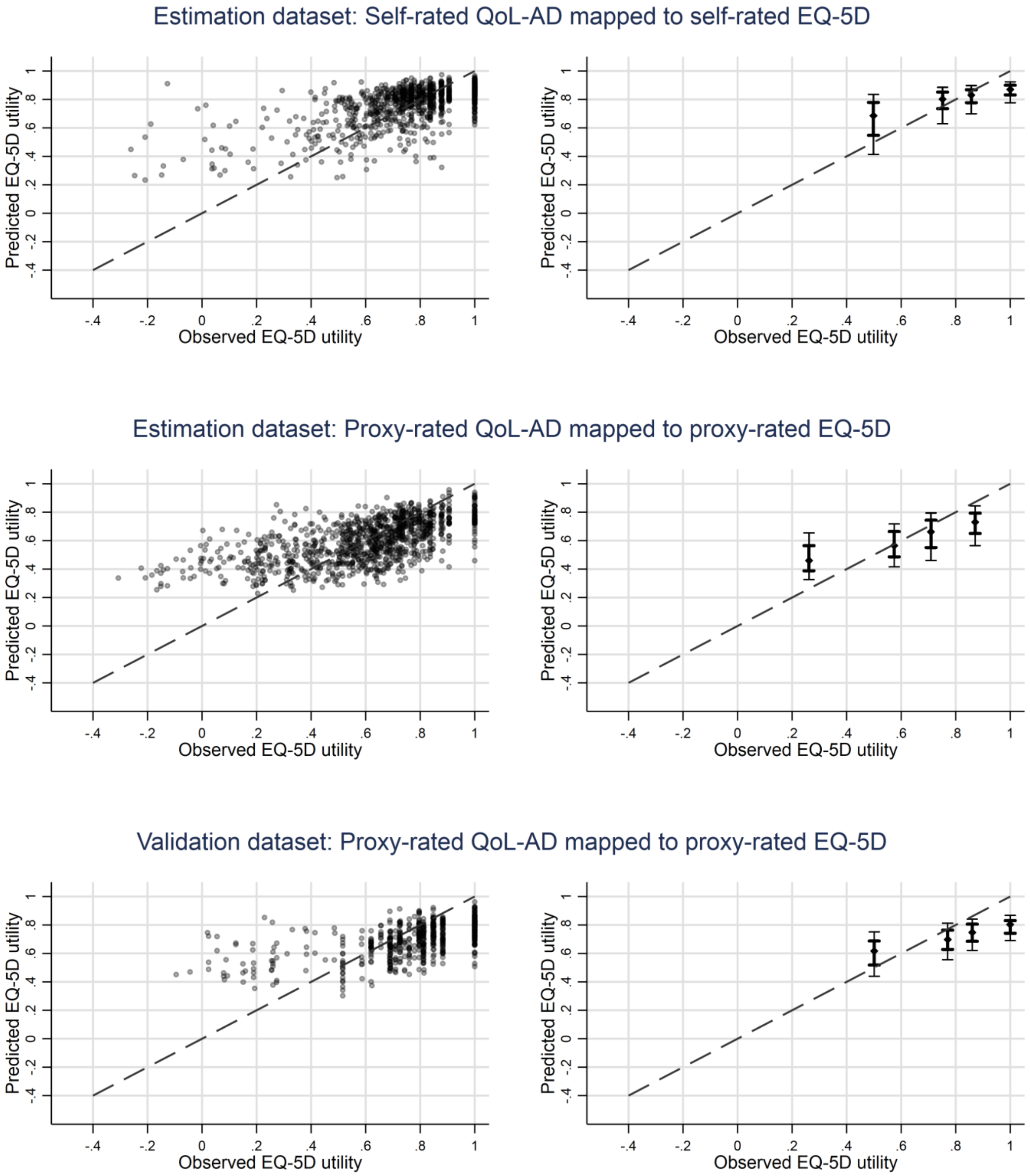

Supplemental Figure 2c: Prediction accuracy of the mapping models using Tobit

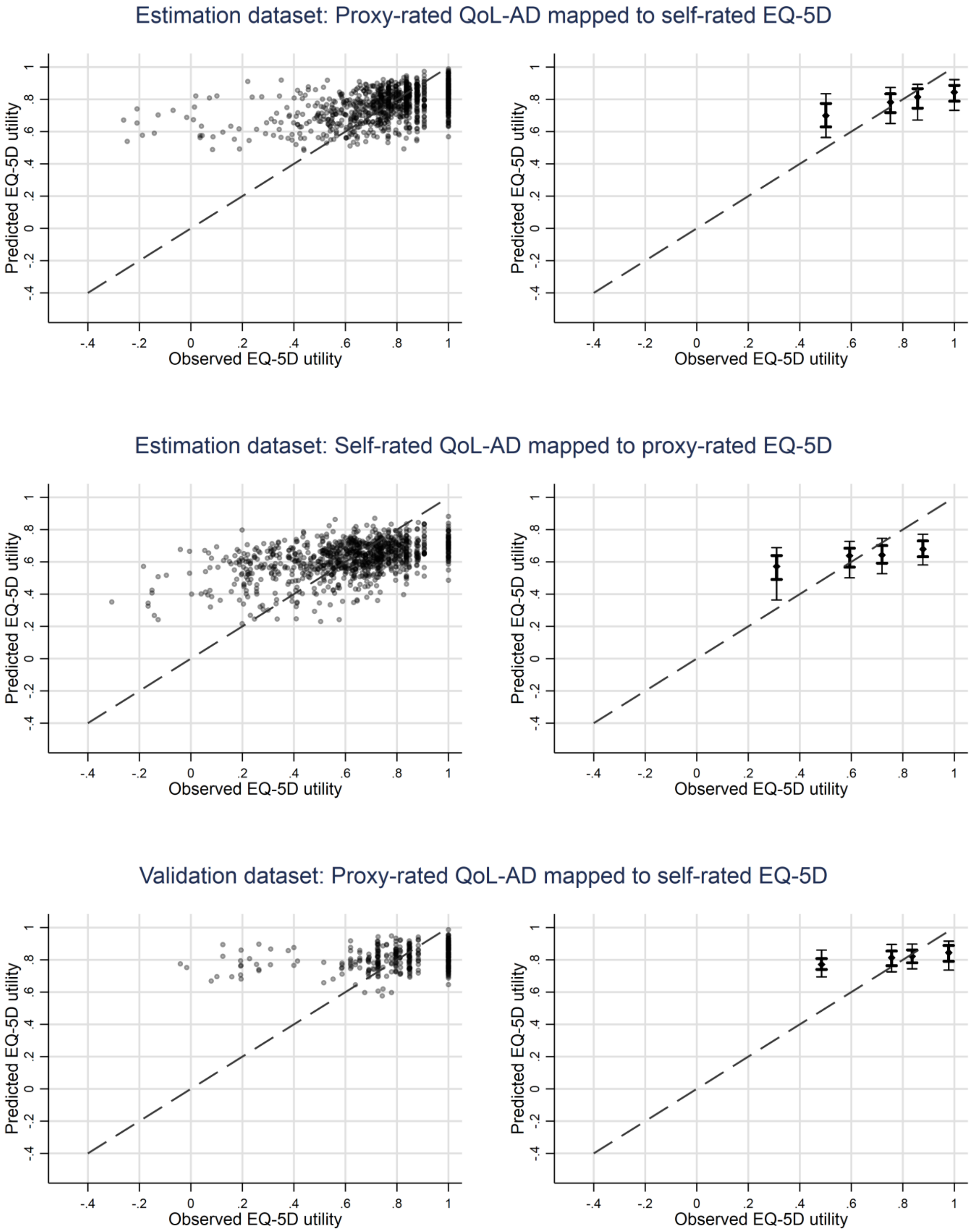

Supplemental Table 6: Assessment of preferred mlogit model across different centiles of QoL-AD scores

			Observed QoL-AD falls into					
			Lowest quartile	IQR	Highest quartile		< median	≥ median
Actifcare dataset	Self-rated QoL-AD → Self-rated EQ-5D	RMSE	0.1570	0.1336	0.1146		0.1470	0.1223
		MAE	0.1213	0.1072	0.0925		0.1153	0.0981
	Proxy- rated QoL-AD → Self-rated EQ-5D	RMSE	0.1792	0.1571	0.1250		0.1671	0.1493
		MAE	0.1406	0.1185	0.0984		0.1291	0.1133
	Self-rated QoL-AD → Proxy-rated EQ-5D	RMSE	0.1925	0.1895	0.1884		0.1895	0.1900
		MAE	0.1516	0.1481	0.1514		0.1487	0.1509
	Proxy- rated QoL-AD → Proxy-rated EQ-5D	RMSE	0.2101	0.1772	0.1454		0.2002	0.1630
		MAE	0.1657	0.1366	0.1097		0.1581	0.1227
Validation dataset	Proxy-rated QoL-AD → Self-rated EQ-5D	RMSE	0.2513	0.1879	0.1810		0.2265	0.1799
		MAE	0.1783	0.1426	0.1311		0.1624	0.1195
	Proxy-rated QoL-AD → Proxy-rated EQ-5D	RMSE	0.2262	0.2030	0.1592		0.2194	0.1739
		MAE	0.1772	0.1593	0.1330		0.1710	0.1417

Supplemental Figure 3: Probability of predicting each response level for a given observed response to the EQ-5D-5L items

Supplemental Figure 3.1.1: Probability of predicting each response level for a given observed response to EQ-5D-5L item 1: self-rated QoL-AD mapped to self-rated EQ-5D-5L

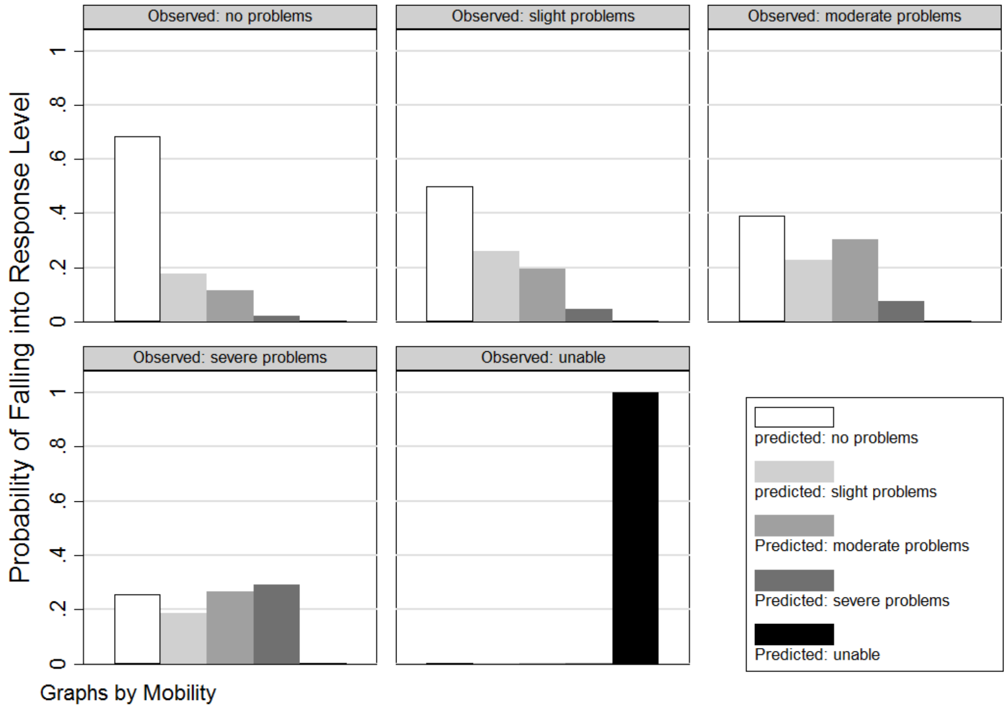

Supplemental Figure 3.1.2: Probability of predicting each response level for a given observed response to EQ-5D-5L item 2 - self-rated QoL-AD mapped to self-rated EQ-5D-5L

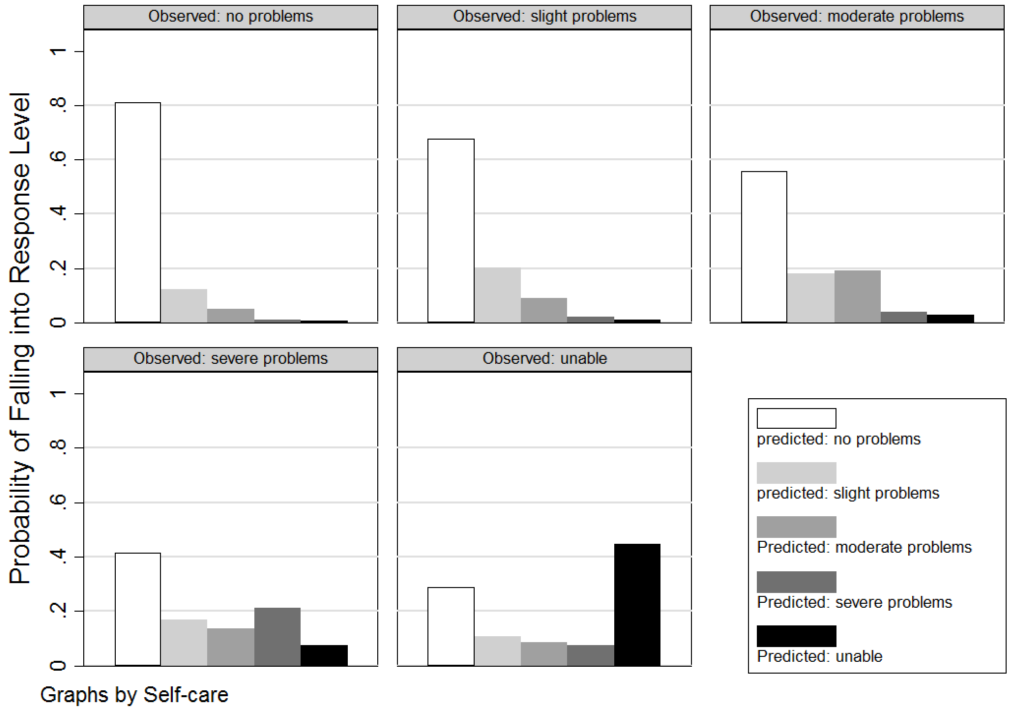

Supplemental Figure 3.1.3: Probability of predicting each response level for a given observed response to EQ-5D-5L item 3 - self-rated QoL-AD mapped to self-rated EQ-5D-5L

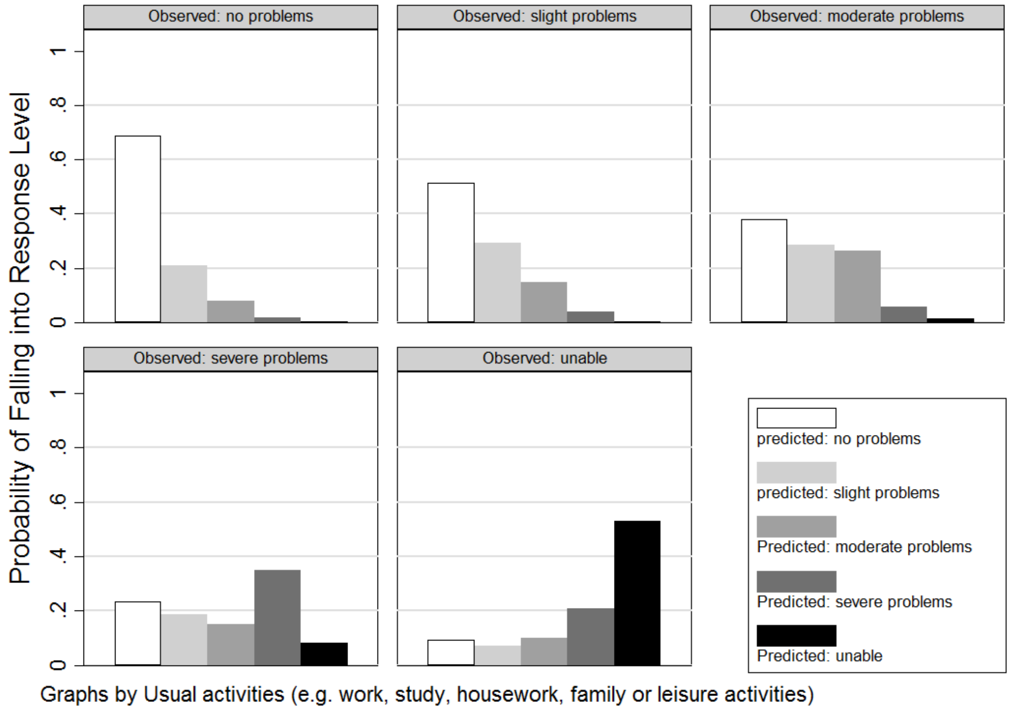

Supplemental Figure 3.1.4: Probability of predicting each response level for a given observed response to EQ-5D-5L item 4 - self-rated QoL-AD mapped to self-rated EQ-5D-5L

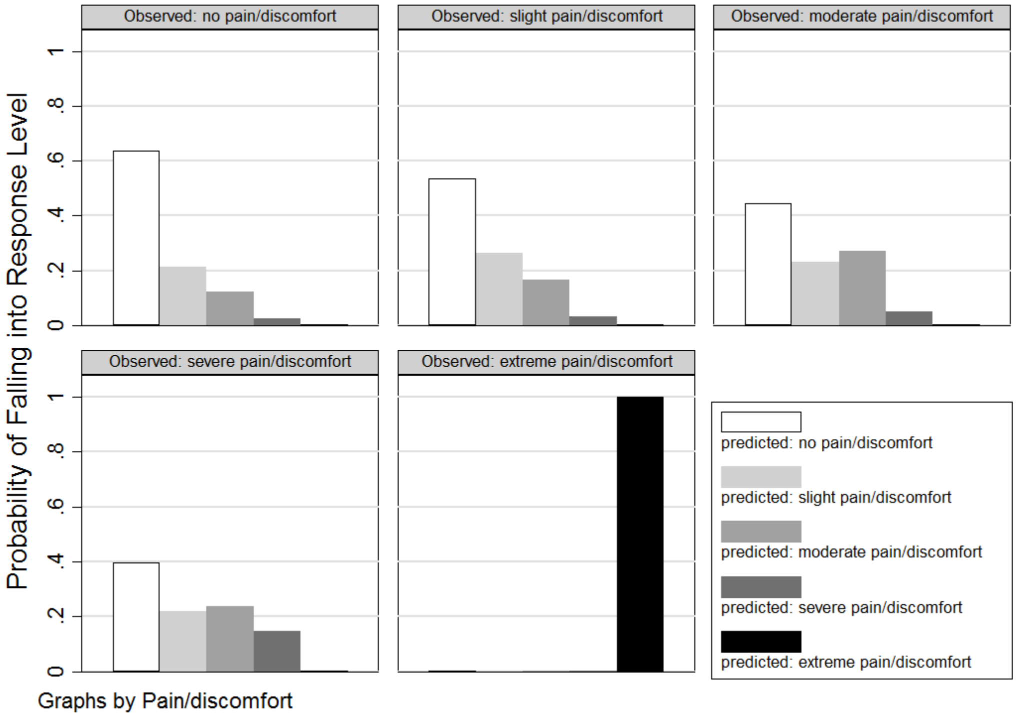

Supplemental Figure 3.1.5: Probability of predicting each response level for a given observed response to EQ-5D-5L item 5 - self-rated QoL-AD mapped to self-rated EQ-5D-5L

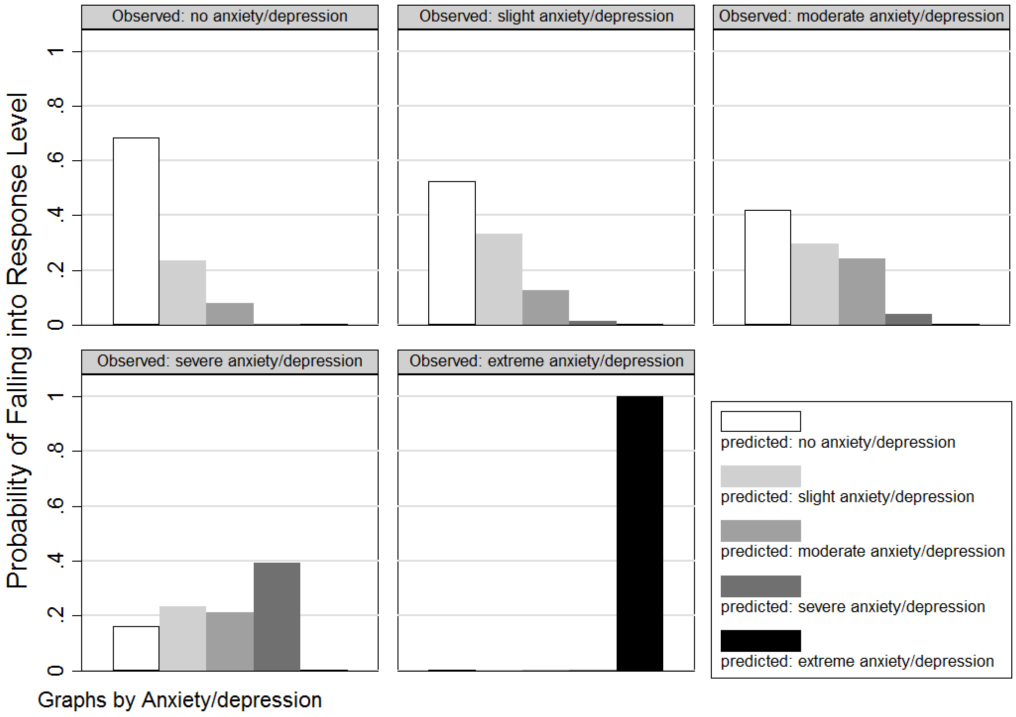

Supplemental Figure 3.2.1: Probability of predicting each response level for a given observed response to EQ-5D-5L item 1 - proxy-rated QoL-AD mapped to self-rated EQ-5D-5L

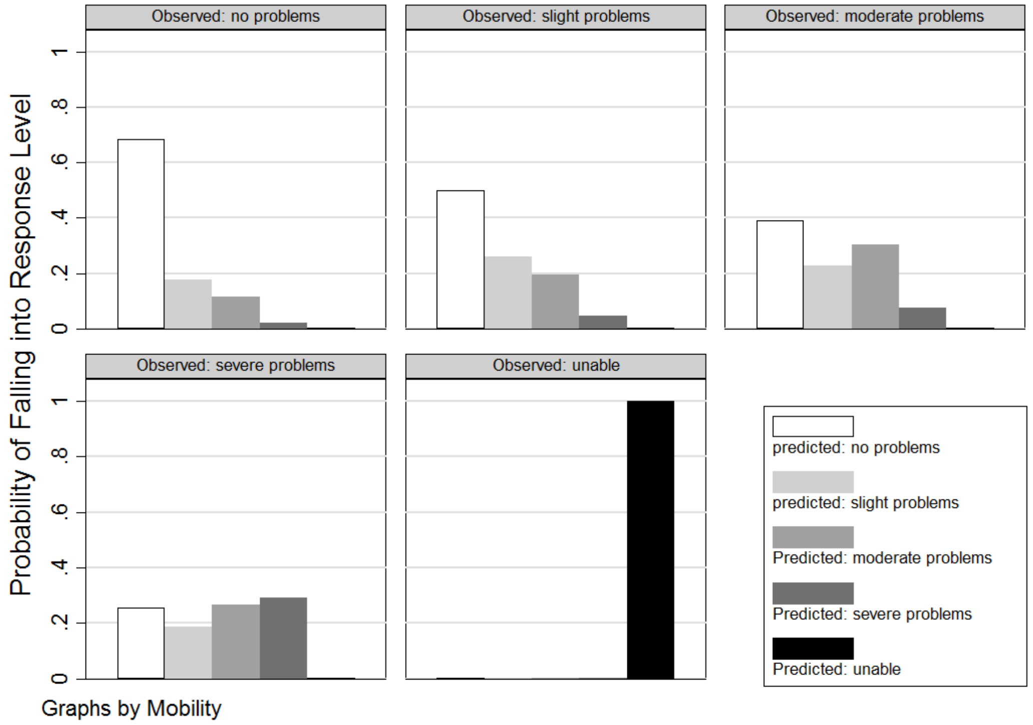

Supplemental Figure 3.2.2: Probability of predicting each response level for a given observed response to EQ-5D-5L item 2 - proxy-rated QoL-AD mapped to self-rated EQ-5D-5L

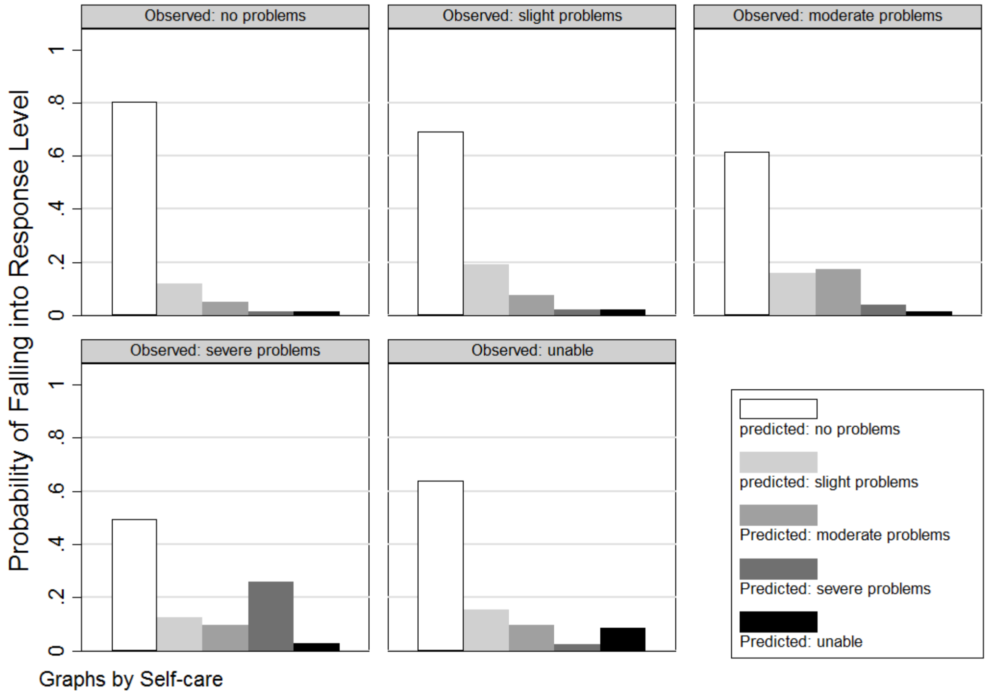

Supplemental Figure 3.2.3: Probability of predicting each response level for a given observed response to EQ-5D-5L item 3 - proxy-rated QoL-AD mapped to self-rated EQ-5D-5L

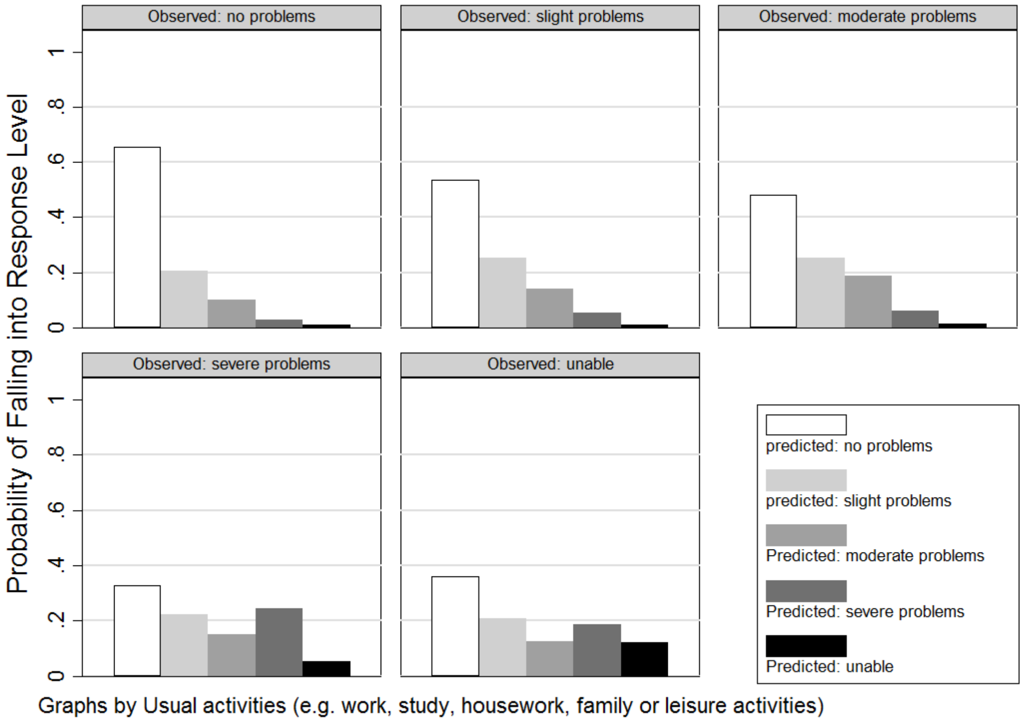

Supplemental Figure 3.2.4: Probability of predicting each response level for a given observed response to EQ-5D-5L item 4 - proxy-rated QoL-AD mapped to self-rated EQ-5D-5L

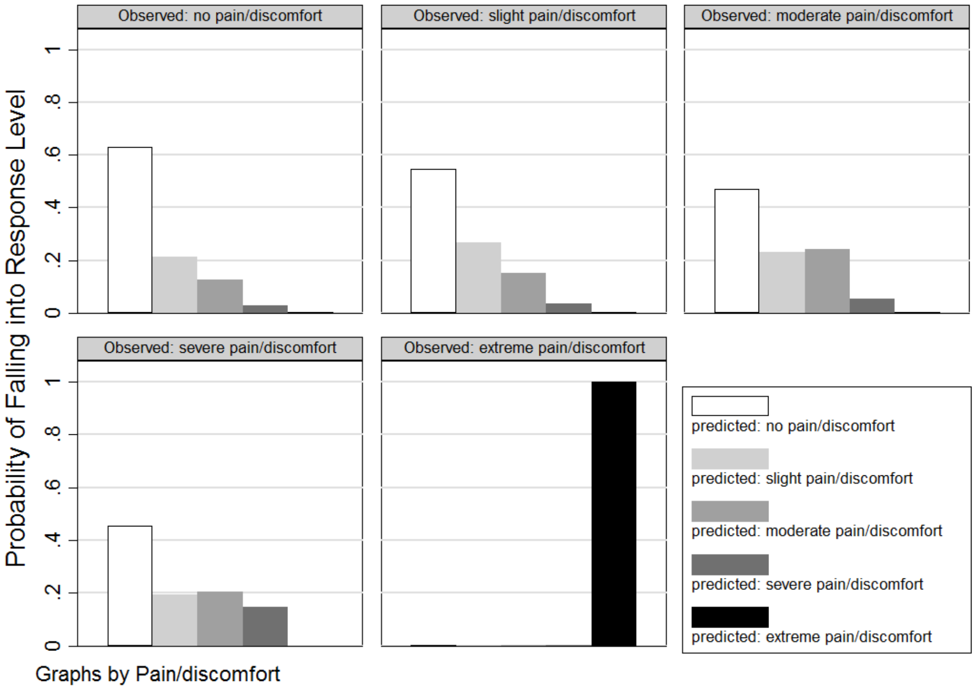

Supplemental Figure 3.2.5: Probability of predicting each response level for a given observed response to EQ-5D-5L item 5 - proxy-rated QoL-AD mapped to self-rated EQ-5D-5L

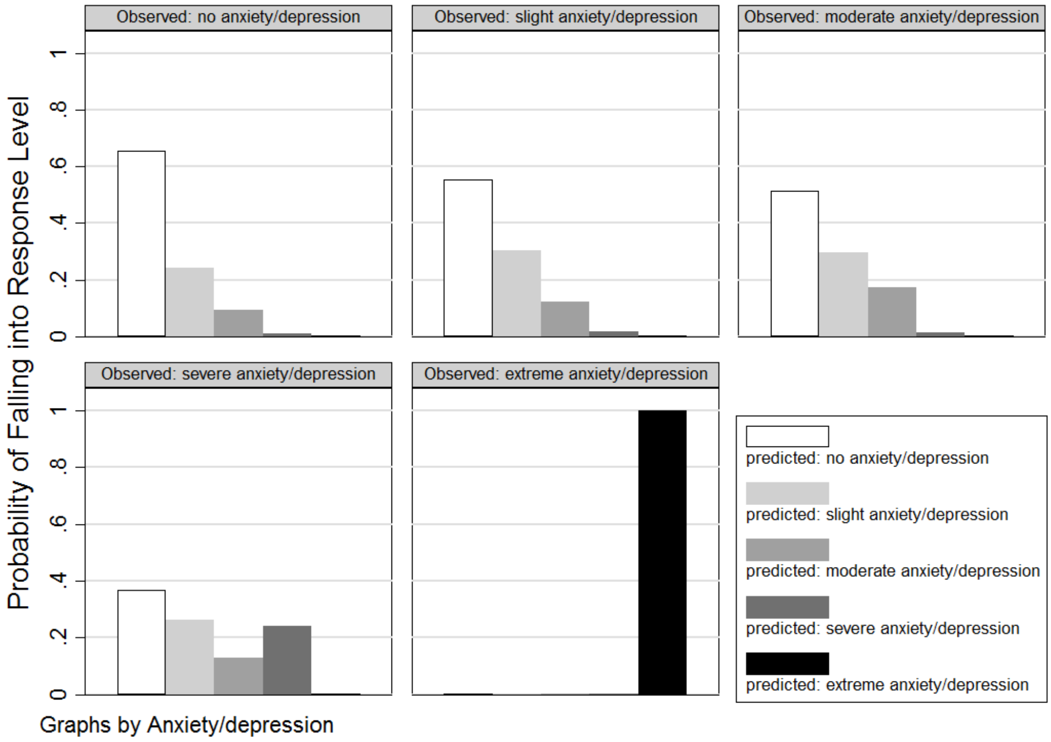

Supplemental Figure 3.3.1: Probability of predicting each response level for a given observed response to EQ-5D-5L item 1 - self-rated QoL-AD mapped to proxy-rated EQ-5D-5L

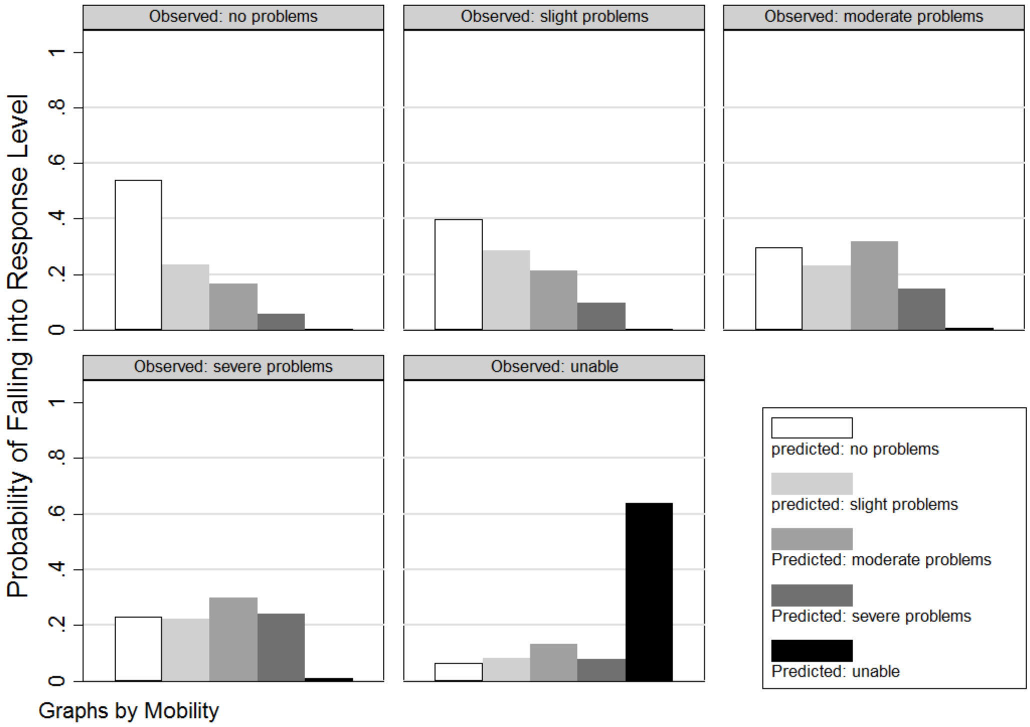

Supplemental Figure 3.3.2: Probability of predicting each response level for a given observed response to EQ-5D-5L item 2 - self-rated QoL-AD mapped to proxy-rated EQ-5D-5L

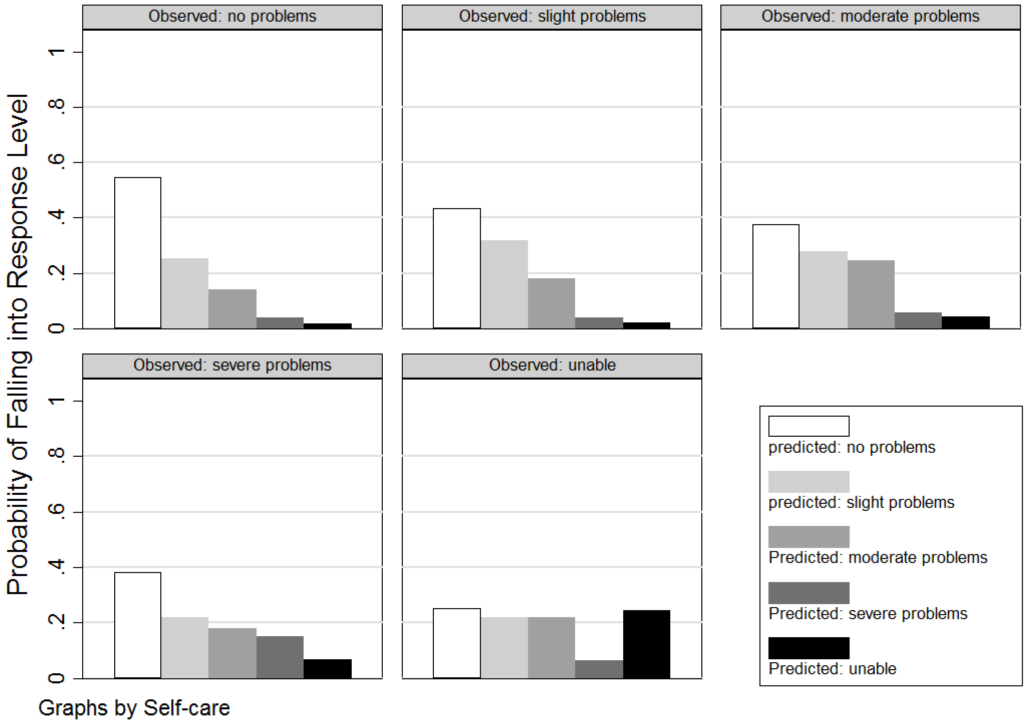

Supplemental Figure 3.3.3: Probability of predicting each response level for a given observed response to EQ-5D-5L item 3 - self-rated QoL-AD mapped to proxy-rated EQ-5D-5L

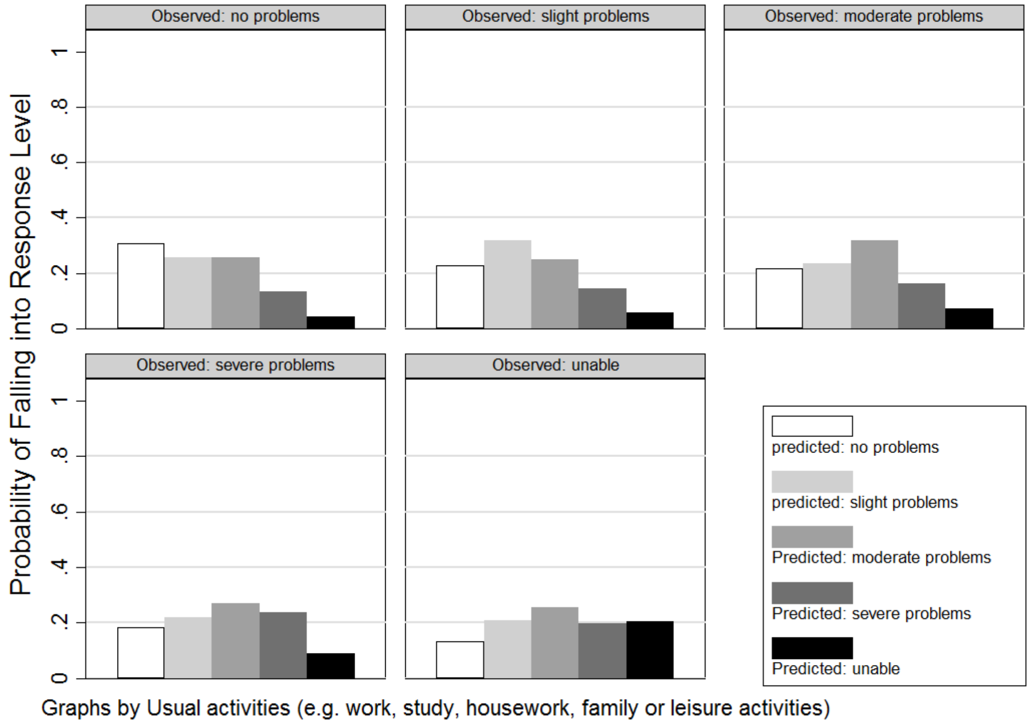

Supplemental Figure 3.3.4: Probability of predicting each response level for a given observed response to EQ-5D-5L item 4 - self-rated QoL-AD mapped to proxy-rated EQ-5D-5L

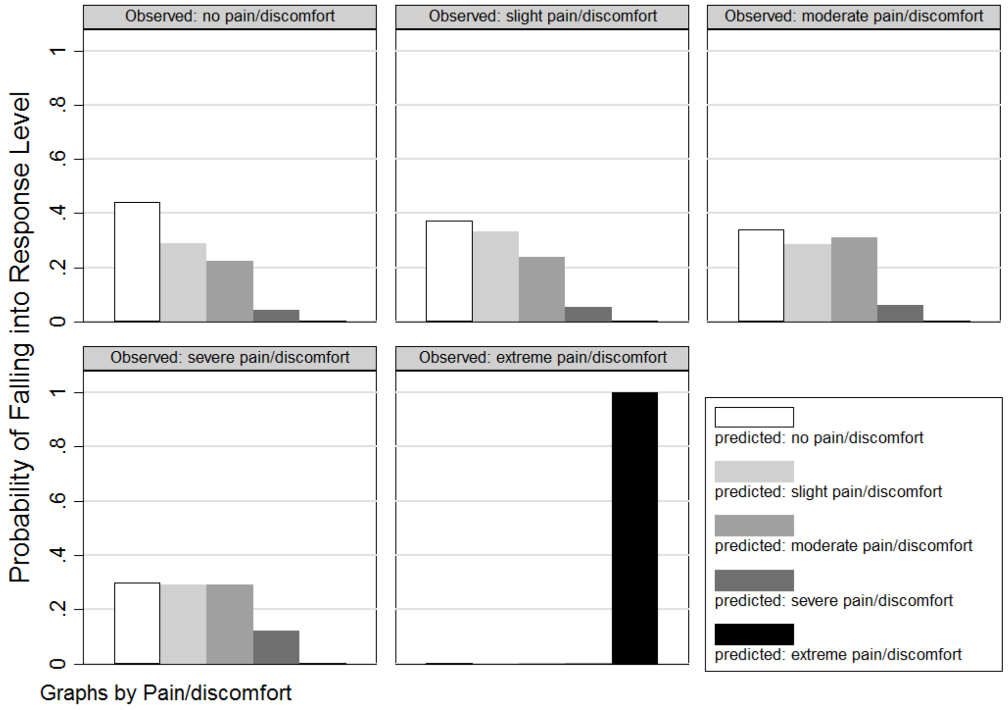

Supplemental Figure 3.3.5: Probability of predicting each response level for a given observed response to EQ-5D-5L item 5 - self-rated QoL-AD mapped to proxy-rated EQ-5D-5L

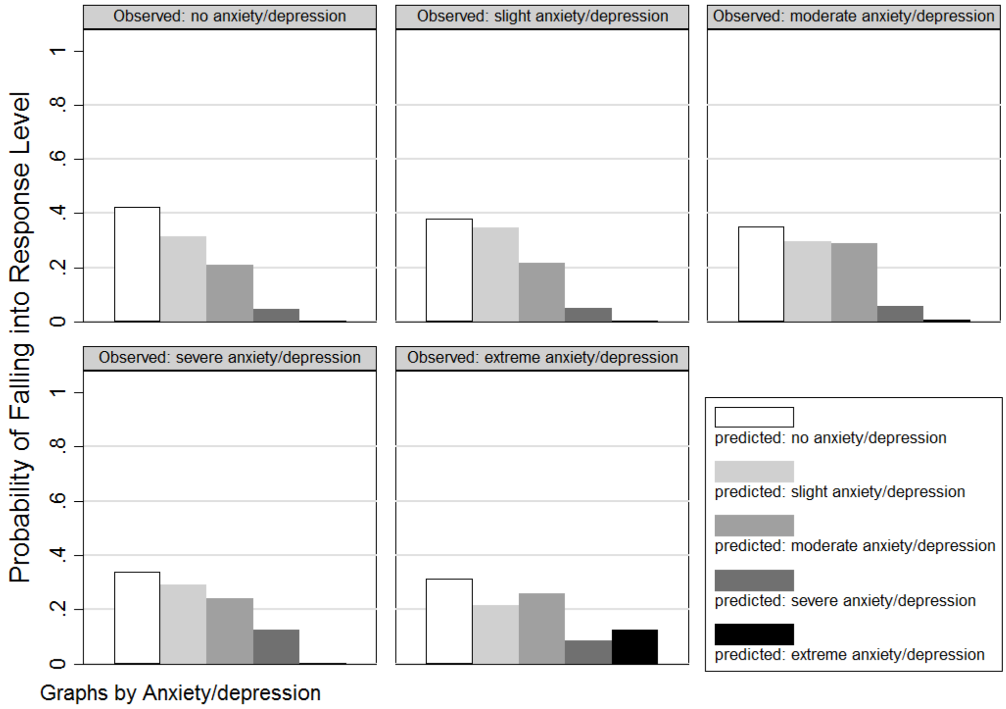

Supplemental Figure 3.4.1: Probability of predicting each response level for a given observed response to EQ-5D-5L item 1 - proxy-rated QoL-AD mapped to proxy-rated EQ-5D-5L

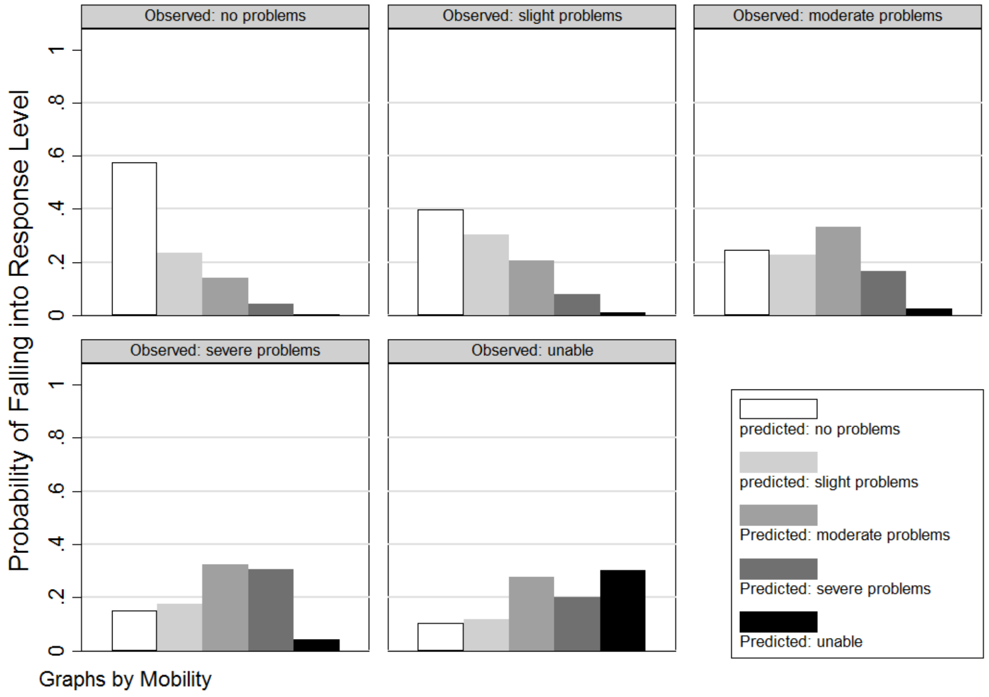

Supplemental Figure 3.4.2: Probability of predicting each response level for a given observed response to EQ-5D-5L item 2 - proxy-rated QoL-AD mapped to proxy-rated EQ-5D-5L

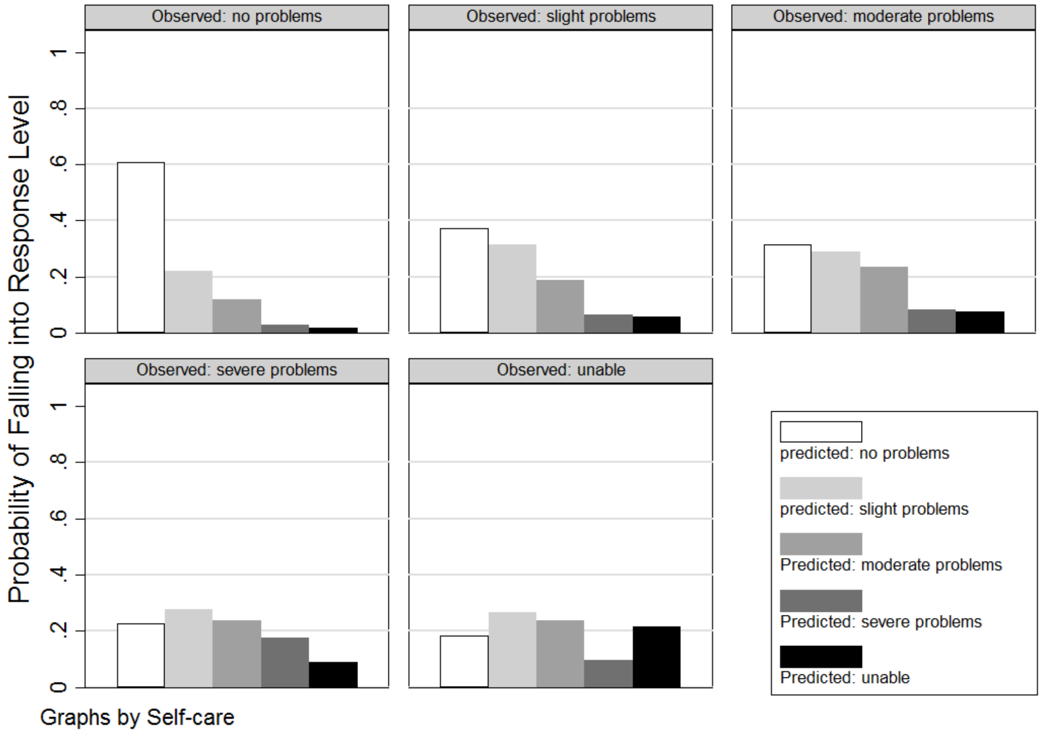

Supplemental Figure 3.4.3: Probability of predicting each response level for a given observed response to EQ-5D-5L item 3 - proxy-rated QoL-AD mapped to proxy-rated EQ-5D-5L

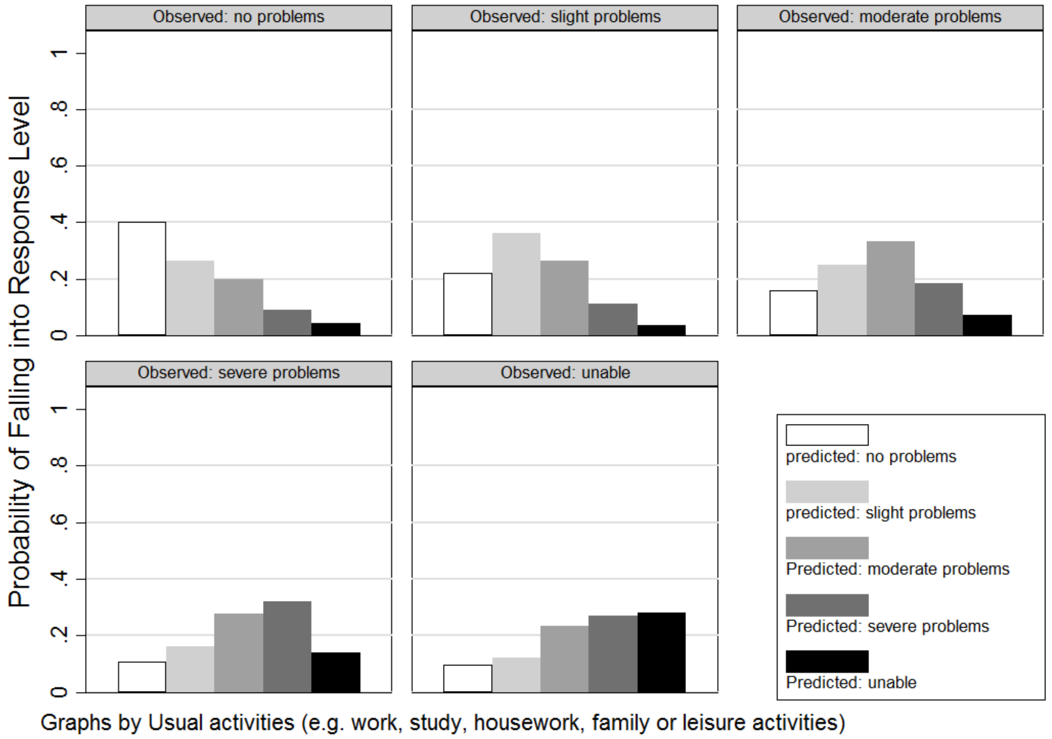

Supplemental Figure 3.4.4: Probability of predicting each response level for a given observed response to EQ-5D-5L item 4 - proxy-rated QoL-AD mapped to proxy-rated EQ-5D-5L

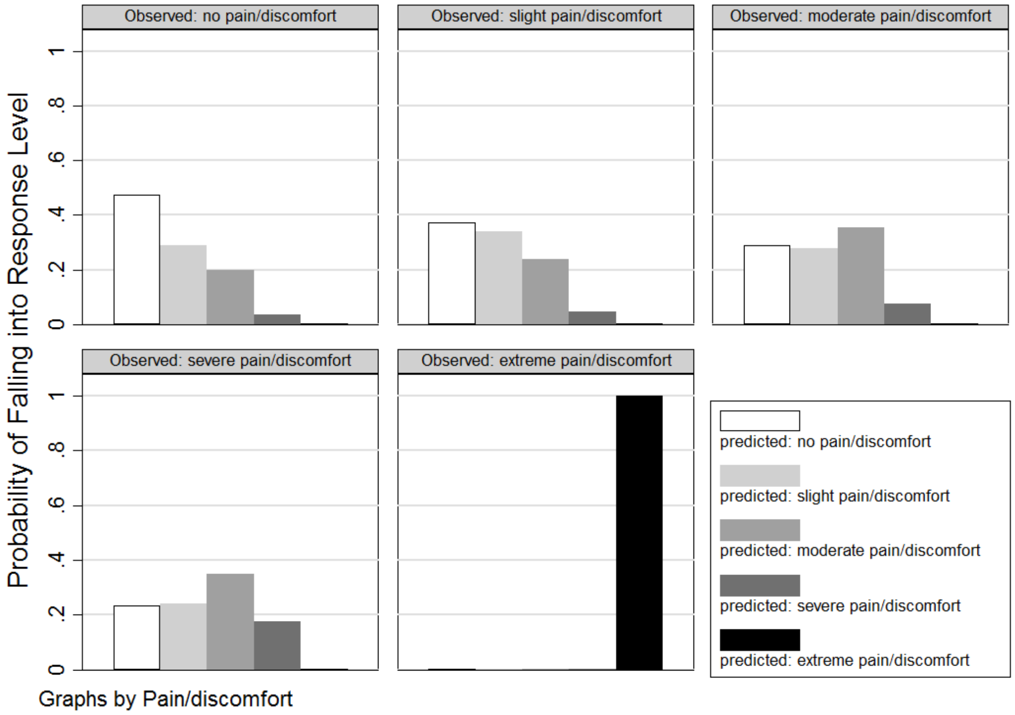

Supplemental Figure 3.4.5: Probability of predicting each response level for a given observed response to EQ-5D-5L item 5 - proxy-rated QoL-AD mapped to proxy-rated EQ-5D-5L

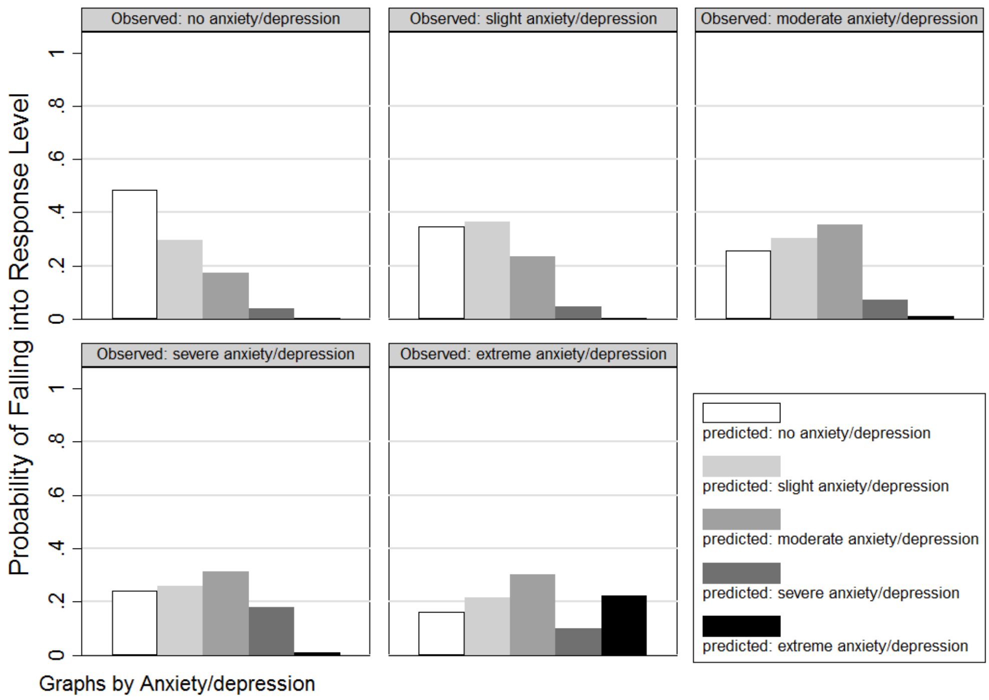

Supplemental Table 7: Mean and range of predicted EQ-5D utilities for observed QoL-AD scores

Observed QoL-AD score*	Self-rated QoL-AD → Self-rated EQ-5D<br>Mean (min, max) N**	Proxy-rated QoL-AD → Self-rated EQ-5D<br>Mean (min, max) N**	Self-rated QoL-AD → Proxy-rated EQ-5D<br>Mean (min, max) N**	Proxy-rated QoL-AD → Proxy-rated EQ-5D<br>Mean (min, max) N**
16.3	0.121 (0.008, 0.234) N=2	0.678 (0.643, 0.707) N=4	0.253 (0.078, 0.428) N=2	0.365 (0.240, 0.516) N=4
24.9	0.632 (0.429, 0.780) N=19	0.730 (0.460, 0.900) N=45	0.547 (0.299, 0.686) N=19	0.514 (0.054, 0.7478) N=50
30.3	0.714 (0.357, 0.848) N=51	0.773 (0.520, 0.902) N=56	0.607 (0.277, 0.742) N=51	0.587 (0.214, 0.788) N=59
32.5	0.727 (0.098, 0.895) N=54	0.757 (0.138, 0.906) N=54	0.122 (0.048, 0.731) N=55	0.640 (0.213, 0.764) N=57
40.0	0.842 (0.451, 0.919) N=63	0.873 (0.785, 0.956) N=23	0.636 (0.058, 0.821) N=63	0.778 (0.594, 0.869) N=24
45.5	0.892 (0.820, 0.972) N=14	0.931 (0.891, 0.970) N=2	0.741 (0.633, 0.840) N=14	0.845 (0.820, 0.869) N=2
52.0	0.965 (0.961, 0.968) N=2	n/a	0.822 (0.815, 0.830) N=2	n/a

This table shows the mean and ranges of the predicted EQ-5D utilities for selected observed QoL-AD scores. Ranges are given, as different combinations of answers to individual items on the QoL-AD can lead to the same overall QoL-AD score, but may nevertheless have different utility values. In fact, predicted utilities may also differ if identical answers to all QoL-AD items were observed, because the predicted utility is also dependent on age and sex.

The selected observed QoL-AD scores are a representative range of observed scores.

Supplemental Figure 4: Range of predicted EQ-5D utilities for observed QoL-AD scores

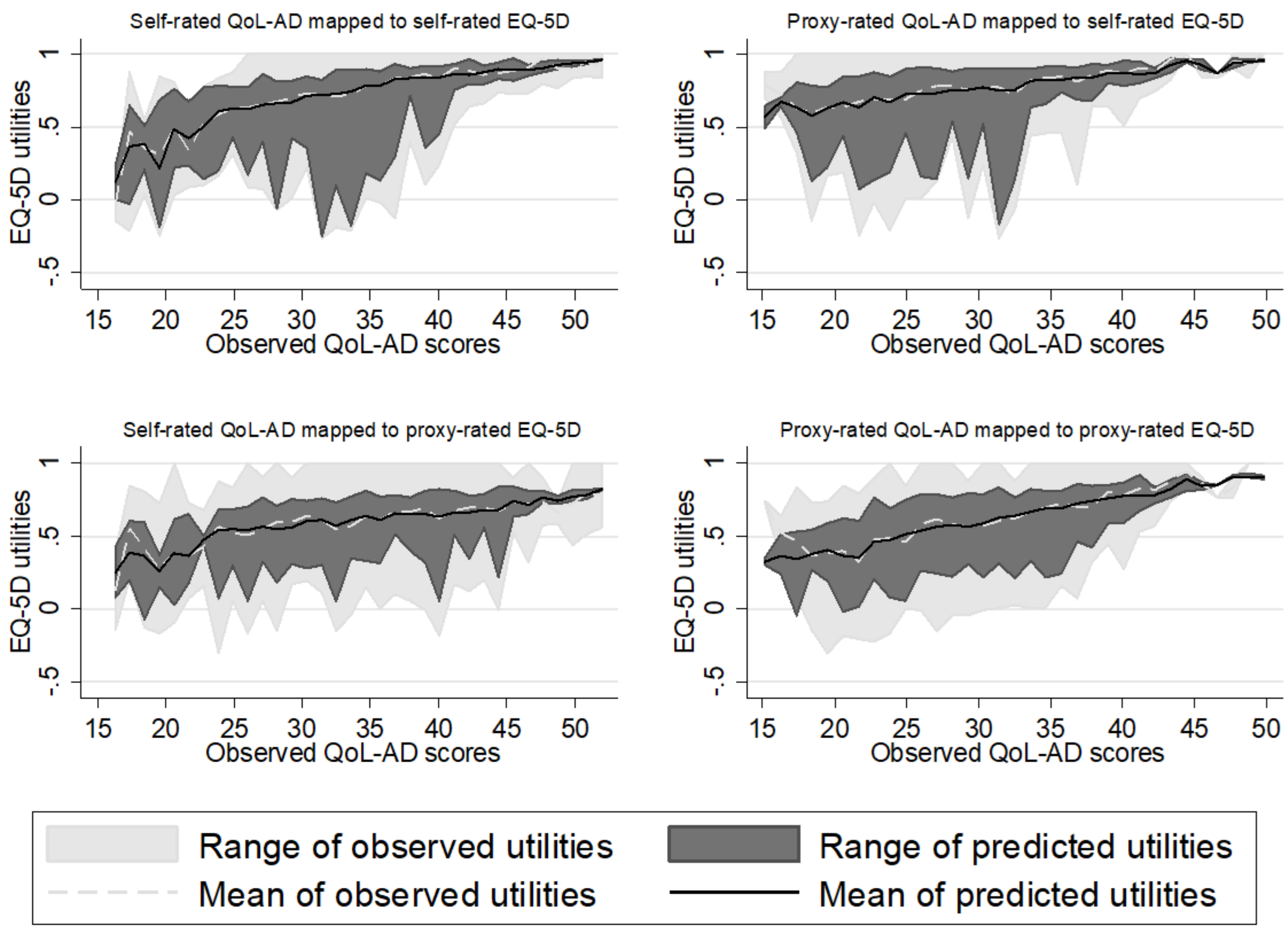

Instructions for the Stata ado-file to map the QoL-AD to the EQ-5D-5L

This document describes how the **map_qolad_to_eq5d5l** Stata ado file is used to obtain EQ-5D-5L utilities mapped from the Quality of Life Alzheimer's Disease Scale (QoL-AD), using either a response mapping approach based on an mlogit model, or a direct mapping approach, based on a Tobit model.

The downloadable material contains files containing the regression coefficients for the different mapping scenarios, as well as the Stata ado file.
These files need to be saved before the mapping program can be run, and Stata needs to be informed about the location of the ado file using the *sysdir set* command (i.e. *sysdir set personal "C:\StataAdoFiles"*).

Command syntax
The syntax for the *map_qolad_to_eq5d5l* command is as follows:
map_qolad_to_eq5d5l, qolad() sex() age() scenario() item7() model() coeffs()

Within the brackets, the following information needs to be specified:

**qolad**	All 13 QoL-AD items need to be listed. If item 7 is not available the dataset, this variable needs to be created as a constant.<br>The items need to be listed in the correct order (i.e. starting with item 1, and proceeding in increasing order to item 13).<br>The QoL-AD data need to be coded as follows: 1=Poor, 2=Fair, 3=Good, 4=Excellent
**sex**	The variable specifying the gender of the person with dementia needs to be listed. This variable needs to be coded 1 for male and 0 for female.
**age**	The variable specifying the age of the person with dementia needs to be listed. For longitudinal data, this should be the age at the time the relevant data were collected.
**scenario**	Specify which mapping scenario should be performed. Choose from:<br>'SelfEQ_SelfQOL' for mapping self-reported QoL-AD to self-reported EQ-5D<br>'ProxyEQ_ProxyQOL' for mapping proxy-reported QoL-AD to proxy-reported EQ-5D<br>'ProxyEQ_SelfQOL' for mapping self-reported QoL-AD to proxy-reported EQ-5D<br>'SelfEQ_ProxyQOL' for mapping proxy-reported QoL-AD to self-reported EQ-5D
**item7**	Specify if the mapping should be performed excluding QoL-AD item 7 (use 'ExcludingItem7') or including QoL-AD item 7 (use 'IncludingItem7')
**model**	Specify mlogit or tobit
**coeff**	The location of the Stata data files containing the regression coefficients for the different mapping scenarios needs to be listed here. The file path needs to be entered without quotation marks.
**dataset**	The location and name of the dataset in which the QoL-AD should be mapped to the EQ-5D needs to be specified. Please note that this program opens a new dataset and will close any datasets currently in use. Please ensure that all data are saved before the mapping map_qolad_to_eq5d5l program is run.

mlogit mapping:
The program generates 26 new variables. 25 of those estimate the probability that a participant will fall into each of the 5 levels for each of the 5 EQ-5D-5L items. Specifically, mob_p1 indicates the probability that the participant falls into the first level of the mobility item ("I have no problems in walking about"), and pa_p5 indicates the probability that a participant falls into the 5th level of the pain item (i.e. "I have extreme pain or discomfort"). 'mob', 'sc', 'ac', 'pa' and 'ad' are used to record information on the mobility, self-care, usual activities, pain/ discomfort and anxiety/ depression items respectively. 'p1' to 'p5' are used to indicate levels 1 ("no problems") to 5 ("unable to" or "extreme problems"). 'eq5d5l_m' contains the EQ-5D-5D utility based on the UK value set (crosswalk to 3L value set, van Hout, 2012). Other country-specific value sets can be derived from the probabilities.
When the Tobit mapping algorithm is used, a single new variable (eq5d5l_t), is created EQ-5D-5D utility based on the UK value set (crosswalk to 3L value set, van Hout, 2012.

Note: The mapping algorithm is currently available in Stata only. We would be very happy to cooperate with other researchers who wish to write code for implementation in SAS, R or other programs.
